# Thiorphan reprograms neurons to promote functional recovery after spinal cord injury

**DOI:** 10.1038/s41586-025-09647-y

**Published:** 2025-10-29

**Authors:** E. A. van Niekerk, C. Marques de Freria, B. O. Mancarci, K. Groeniger, D. Kulinich, T. Riley, R. Kawaguchi, S. Okawa, T. Vokes, E. S. Rosenzweig, E. Sinopoulou, M. J. Castle, R. Huie, A. R. Ferguson, N. Kfoury-Beaumont, A. Khalessi, P. Pavlidis, M. H. Tuszynski

**Affiliations:** 1 Department of Neurosciences, University of California San Diego, La Jolla, CA USA; 2 Department of Psychiatry, Michael Smith Laboratories, Djavad Mowafaghian Centre for Brain Health, University of British Columbia, Vancouver, British Columbia Canada; 3 David Geffen School of Medicine, University of California Los Angeles, Los Angeles, CA USA; 4 Vascular Medicine Institute, University of Pittsburgh, Pittsburgh, PA USA; 5 Brain and Spinal Injury Center, University of California, San Francisco, CA USA; 6 San Francisco Veterans Affairs Medical Center, San Francisco, CA USA; 7 Department of Neurological Surgery, University of California San Diego, San Diego, CA USA; 8 Veterans Administration Medical Center, San Diego, CA USA

**Keywords:** Spinal cord injury, Neural stem cells

## Abstract

We previously identified an embryonic shift in the corticospinal motor neuronal transcriptome after spinal cord injury associated with successful axonal regeneration^1^. Exploiting this transcriptional regenerative ‘signature’, here we used in silico screens to identify small molecules that generate similar shifts in the transcriptome, and identified thiorphan—a neutral endopeptidase inhibitor—as a lead candidate. In a new adult motor cortex neuronal in vitro screen^2^, thiorphan increased neurite outgrowth 1.8-fold (*P* < 0.001). We then infused thiorphan into the central nervous system beginning 2 weeks after severe C5 spinal cord contusions and, when combined with a neural stem cell graft, thiorphan elicited significant improvements in forelimb function (*P* < 0.005) and corticospinal regeneration (*P* < 0.05). Extending clinical relevance, thiorphan significantly increased neurite outgrowth in primary cortical neuronal cultures from a 56-year-old human. These findings represent a new path for drug discovery, starting from in silico screens to proof-of-concept in adult human brain cultures.

## Abstract (web summary)

Thiorphan is identified as a new and effective drug treatment for spinal cord injury that can be adopted across nervous system disease states in the development of new therapies for human neurological disorders.

## Main

There are no effective therapies for promoting neural repair after spinal cord injury (SCI), which represents a disorder of great unmet medical need. We reported recently that the corticospinal projection—the most important motor system for voluntary movement in humans—undergoes regression to an embryonic transcriptional state after adult SCI in mice^1^; in this state, the corticospinal axon regenerates into a neural stem cell graft placed in the site of the injury.

Taking advantage of established bioinformatics approaches, in silico screens and medium-throughput model systems of adult motor cortex neuronal cultures^2^, we sought to identify drugs and small molecules that replicated the regenerative transcriptional state of the corticospinal neuron. First, we compared the injured and regenerating corticospinal transcriptome with a publicly available database of several thousand drugs and small molecules already tested in humans—the Connectivity Map^3^ (CMap). This in silico screen identified several compounds that mimicked, to varying degrees, the corticospinal regenerating transcriptome. We then tested these lead compounds in a newly developed in vitro screen in cultures of adult cortical motor neurons. A lead small molecule candidate, thiorphan (253 Da), known primarily as a neutral endopeptidase inhibitor, exhibited the greatest efficacy in supporting total neurite outgrowth and longest neurite length in this medium-throughput screen. We then tested the ability of thiorphan to support axonal regeneration and functional recovery after severe, bilateral mid-cervical SCI in adult rats when delivered 2 weeks after SCI—a clinically relevant time point for intervention. Notably, thiorphan treatment combined with a neural progenitor cell (NPC) graft significantly improved functional recovery after SCI. Moreover, thiorphan administration significantly augmented corticospinal axon regeneration into stem cell grafts. Finally, pursuing the true translational potential of thiorphan, we tested its effects on primary cultures of adult cynomolgous monkey motor cortex and 56-year-old human cortex neurons: once again, thiorphan significantly increased neurite growth. Thus, a bioinformatics-driven approach combined with an in vitro screen, in vivo testing and primate in vitro confirmation represent a new pathway for drug discovery. Thiorphan previously underwent human testing for a different disease indication, enhancing its readiness for development as a therapy for SCI.

## A pipeline for drug discovery

**Fig. 1:**
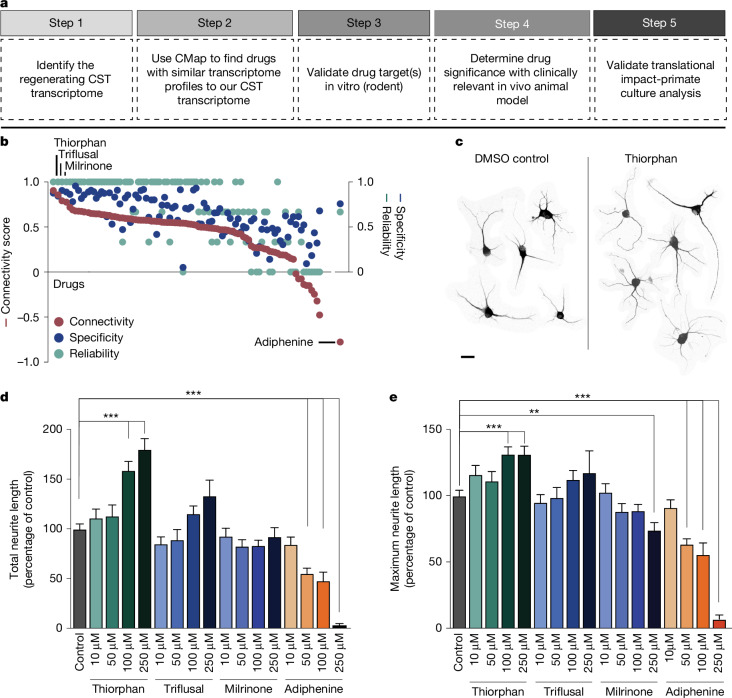
Drug discovery pipeline, in silico analysis and in vitro validation. **a**, Drug discovery pipeline consisting of five steps: (1) creation of a transcriptomic dataset; (2) in silico analysis using CMap; (3) medium-throughput in vitro screen; (4) in vivo testing of lead candidate(s); and (5) in vitro monkey and human validation. CST, corticospinal tract. **b**, In silico analysis shows ranking of compounds in CMap on the basis of connectivity score (left *y* axis), specificity score (blue, right *y* axis) and reliability score (green, right *y* axis) (see text for further detail). **c**, Neurite extension in composite image of cortical neurons treated with DMSO control and thiorphan (250 μM), Tuj1 labelling. **d**,**e**, In vitro screen of top three ‘hits’ from in silico screen, together with predicted negative modulator, adiphenine. Tested in cultures of adult mouse primary cortical neurons for 5 days in vitro, three independent biological replicates were performed on separate days, each using neurons from four adult mice. For each condition, data from all neurons measured across replicates were combined to yield *n* = 200 neurons per condition. Each neuron was spatially separated and analysed as an independent observation. Total neurite outgrowth per cell (Tuj1 labelling) is shown for each condition. Median values: DMSO (82.9); thiorphan (90.7, 99.8, 132.4, 166.1); triflusal (74.3, 61.7, 94.5, 124.5); milrinone (78.9, 77.4, 68.5, 75.5); adiphenine (74.4, 44.5, 31.7, 3) (**d**). Maximum neurite length per cell is shown for each condition. Median values: DMSO (93.3); thiorphan (111.1, 105.4, 122.1, 128); triflusal (84.4, 85, 104.9, 114.3); milrinone (100.6, 81.7, 79, 78.6); adiphenine (87.4, 60, 30.6, 6.23) (**e**). Statistical significance was determined by two-tailed Student’s *t*-test (**P < 0.01, ***P < 0.001). Error bars ± s.e.m. (**d**,**e**). Scale bar, 25 μm (**c**).

We adopted a five-step pipeline to identify candidates for enhancing neural repair with a streamlined path that could lead to human testing (Fig. 1a). Step 1 involves characterizing the transcriptomic state or ‘signature’ of a cell type that exhibits a beneficial biological effect. In this case, we used the transcriptome of corticospinal motor neurons that are capable of regeneration after SCI. We reported previously that, within 2 weeks of SCI, the corticospinal neuron undergoes transient transcriptional reversion to an embryonic state—a state in which it is capable of regenerating^1^. These data are publicly available. In Step 2, we perform an in silico screen to compare the regenerating corticospinal transcriptome with shifts in the transcriptome generated by more than 1,300 small molecules or compounds in the Broad CMap (https://bioconductor.org/packages/release/data/experiment/html/ConnectivityMap.html). Drugs and small molecules in CMap have been exposed to various cell lines in vitro, and effects of these compounds on the transcriptome of the cultured cells have been characterized^3^. Thus, we searched CMap for compounds that generated transcriptomic shifts that parallel the state of regenerating corticospinal neurons. In Step 3, we took the lead candidates from the in silico screen and applied them to a medium-throughput in vitro assay consisting of cultures of adult motor cortex neurons; this screen became available only recently^2^ and provides what could be the most useful in vitro system for predicting potential in vivo effects in the mature nervous system, because the screen consists of adult rather than embryonic or postnatal neurons. To date, in vitro cellular screens of the nervous system consisted of either embryonic or early postnatal neuronal cultures, which may not accurately reflect responses of adult central nervous system (CNS) neurons. Immortalized neural cell lines^4^ suffer from similar limitations. Although adult dorsal root ganglia (DRG) neurons can be cultured routinely, these cells exhibit fundamentally different transcriptomes before and after injury^1,5,6^ and, again, might not be accurate tools for predicting therapeutic benefits of candidate therapies for injured brain and spinal cord motor neurons. Induced pluripotent stem cells or neurons induced from other cell types also often exhibit early cellular markers^7^. Cultures of adult neurons therefore may represent the most optimal in vitro screen developed for clinical translation^2^. Step 4 consists of in vivo validation of the top drug arising from the in vitro screen in a model of SCI. Finally, Step 5 consists of validating candidate compounds in cultures of adult primate cortical neurons, including human adult cortex. This step follows rather than precedes in vivo testing because of the scarcity of the primate and human neuronal resource: normal human neurons in particular are available only occasionally from surgical specimens and in our case were obtained from an individual undergoing tumour resection through a cortical window, where pathology confirmed the cortical biopsy was normal tissue.

### Characterizing the transcriptomic state

Step 1 was accomplished in this study by using our previous data describing the transcriptome of the regenerating corticospinal neuron^1^, as referenced above.

### In silico analysis

In silico analysis directly followed the original methodology of Lamb et al.^3^, identifying several compounds that most closely matched the expression profile of the regenerating corticospinal system (Fig. 1b and Supplementary Fig. 1a). A ‘connectivity score’ with a value between +1 and −1 described the similarity between the corticospinal transcriptomic profile and an individual compound’s transcriptomic profile, where +1 was most similar and −1 was least similar to the corticospinal dataset (Fig. 1b). We further calculated a ‘reliability score’ for each compound that takes into account the connectivity score, *P* value, false detection rate (FDR), ‘instance count’ (number of experiments that include the compound), non-null score (the ratio of experiments that have a consistent score based on most experiments) and ‘specificity score’ derived from the distribution of enrichment scores for gene lists from MSigDB; see Methods for a more detailed description of the reliability score. The top compounds that met the criteria of highest enrichment, reliability and specificity scores were considered ‘hits’ in this assay and included: quinostatin (highest enrichment score +0.91 ± 0.07), thiorphan (+0.86 ± 0.11), triflusal (+0.79 ± 0.13) and milrinone (+0.78 ± 0.18), whereas adiphenine was the most negatively correlated (−0.78 ± 0.18; Fig. 1b and Supplementary Fig. 1b). Biological annotation (Methods) indicated that quinostatin inhibits PI3 kinase; thiorphan is a neutral endopeptidase inhibitor; triflusal inhibits nuclear factor κB, phosphodiesterase and COX1, and milrinone is a phosphodiesterase inhibitor (Supplementary Fig. 1a). Quinostatin was not available for purchase and attempts at synthesis did not yield adequate quality compound. Thiorphan has been characterized primarily as a neutral endopeptidase inhibitor targeting neprilysin^8^. It has also been linked to neuroprotective properties in models of perinatal excitotoxic brain lesions^9^ and diabetic retinopathy^10^, and has β-amyloid degrading properties^11^. Thiorphan, milrinone and triflusal were taken forward as positive candidates, and adiphenine was used as a negative control.

### In vitro adult motor cortical culture screen

We assessed the lead candidate compounds in dissociated cultures of the adult (postnatal day 60) mouse motor cortex^2^. An average of 10,000 neurons per single motor cortex were obtained, and we quantified total neurite outgrowth and maximal neurite length per cell 5 days after exposure to the candidate compounds. Several drug doses were applied (Fig. 1d,e), including the predicted negative modifier of corticospinal growth, adiphenine. Significant overall differences in neurite outgrowth were present using various compounds (drug type × dose interaction generalized estimating equation for total neurite outgrowth, Wald chi-square = 60.67, *P* < 0.0001; drug type × dose interaction generalized estimating equation for total maximum neurite length, Wald chi-square = 227.78, *P* < 0.0001). Thiorphan stood out as most effective both in measures of total neurite outgrowth and neurite length: at a dose of 250 μM it increased total neurite outgrowth by 80% compared with controls (post hoc generalized estimating equation, Wald chi-square = 29.69, *P* < 0.001; Fig. 1d,e) and longest neurite length by 30% (post hoc generalized estimating equation, Wald chi-square = 12.62, *P* < 0.001; Fig. 1d,e). The effect of thiorphan was dose dependent, with peak effects occurring at doses between 100 μM and 250 μM. The effect of the next candidate drug for enhancing corticospinal regeneration, triflusal, was more modest, with an increase in neurite outgrowth of 30% at its peak dose of 250 μM with no significant change in longest neurite length; none of these changes reached statistical significance (Fig. 1e). The third candidate in rank order, milrinone, did not significantly influence either total neurite outgrowth or longest neurite length at any dose (Fig. 1d,e). Validating the in silico screening process, the predicted negative regulator of corticospinal growth, adiphenine, strongly and significantly reduced total neurite outgrowth and longest neurite length in a dose-dependent manner (post hoc generalized estimating equation, *P* < 0.001; Fig. 1c–e).

### In vivo testing in a model of SCI

**Fig. 2:**
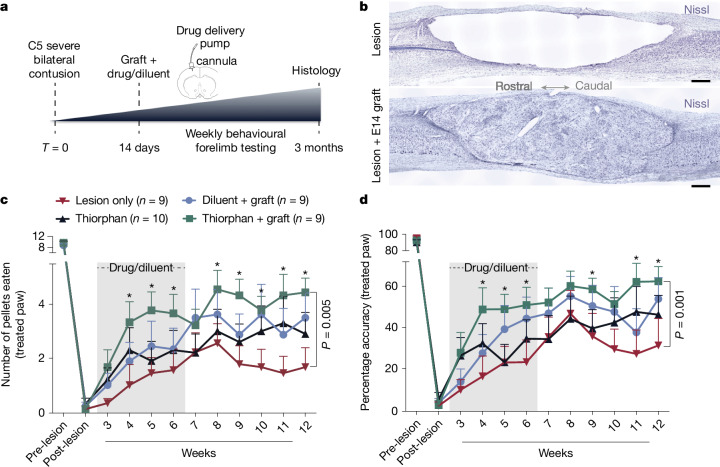
Thiorphan improves functional outcomes after severe C5 bilateral contusion. **a**, In vivo experimental outline of a clinically relevant SCI model. **b**, Nissl stain of a severe C5 bilateral contusion model. Sagittal section, rostral to left. Top, large contusion cavity in an ungrafted animal after 3 months. Bottom, NPC graft filling the lesion cavity and providing substrate for potential corticospinal axon regeneration. **c**, Skilled forelimb successful pellet retrieval with right paw on Montoya staircase^23^ ± s.e.m. Animals that received thiorphan and a substrate for axonal regeneration into the lesion site—an NPC graft—exhibit significant functional recovery over time compared with lesioned controls (*P* = 0.005, group × time interaction, Poisson generalized linear model). Treatment with thiorphan alone also trended towards significance (*P* = 0.14). The lesion model applied in this experiment is the most severe that we have tested, and NPC grafts also exhibited a trend towards better outcomes than lesioned controls but this did not reach statistical significance. Grey shading, period of thiorphan infusion into cortex. **d**, Thiorphan + NPC graft group also exhibits significant recovery of pellet retrieval accuracy in this severe lesion model (*P* = 0.001). Other treated groups trend towards improved outcomes compared with lesioned controls. Accuracy represents the number of pellets eaten divided by the number of pellets displaced plus the number of pellets eaten. *P < 0.05 (**c**,**d**). Scale bar, 500 μm. Source data

Given the success of thiorphan in demonstrating efficacy in the in vitro screen, it advanced to Step 4: in vivo testing in a clinically relevant model of SCI. Adult Fischer 344 rats underwent severe contusive bilateral cervical SCI at the C5 spinal cord segment; mid-cervical lesions are the most common levels of human injury^12^ (Supplementary Fig. 2a,b). We then waited 2 weeks—a clinically relevant time to delay intervention to allow subject recovery from the acute effects of injury (Fig. 2a). Animals were then divided into one of four treatment groups: (1) lesion alone (*n* = 9), (2) thiorphan alone (*n* = 10), (3) diluent + NPC graft (*n* = 9) and (4) thiorphan + NPC graft (*n* = 9). We tested diluent + NPC grafts as a positive control in this lesion model because these cells support axonal regeneration into the lesion site, the formation of new neural relays across the injury site and partial functional recovery^13,14^. We also compared thiorphan alone with NPCs. The combination of thiorphan + NPCs aimed to determine whether effects of both treatments were additive, thereby representing a method of further augmenting recovery after severe cervical SCI. Thiorphan was infused continuously into the left motor cortex because it does not cross the blood–brain barrier. Infusions continued for 4 weeks starting 2 weeks after injury at a dose of 100 mM (0.25 μl h^−1^), based on extrapolation of in vitro dosing to in vivo dosing^15^. An in vivo neprilysin cleavage assay confirmed the bioactivity of intraparenchymal motor cortex infusions of thiorphan over 1, 2 and 4 weeks (Supplementary Fig. 2c). Spinal cord NPC grafts were obtained from Fischer 344 embryonic-day-14 (E14) spinal cords as reported previously^16^ and injected through the dura into the lesion cavity 2 weeks post SCI (Fig. 2b; Methods). Two weeks before euthanasia, the corticospinal projection was traced anterogradely using an AAV9-CAG–Ruby2sm_Flag.

Twelve weeks after SCI, animals treated with thiorphan + NPC grafts exhibited a significant, twofold improvement in forelimb grasping success (number of pellets grasped and eaten) after severe mid-cervical SCI compared with lesioned controls (Poisson generalized linear model, Wald chi-square = 7.92, *P* = 0.005; Fig. 2c). Recipients of thiorphan alone or NPC grafts alone exhibited lesser degrees of forelimb recovery compared with lesioned controls that did not reach statistical significance (Poisson generalized linear model, Wald chi-square = 2.16, *P* = 0.14 comparing thiorphan with lesion alone, and Wald chi-square = 2.07, *P* = 0.15 comparing grafts-only with lesion alone; Fig. 2c). The accuracy of pellet retrieval (percentage of pellets grasped that are eaten) also improved significantly in animals that received thiorphan + NPC grafts (Gamma generalized linear model, Wald chi-square = 10.13, *P* = 0.001) compared with lesioned controls (60% accuracy versus 30% accuracy; Fig. 2d). Thus, as suggested by in silico and subsequent in vitro screens, thiorphan significantly improves functional outcomes when combined with neural stem cell grafts to sites of SCI by enhancing the potency of NPC grafting.

**Fig. 3:**
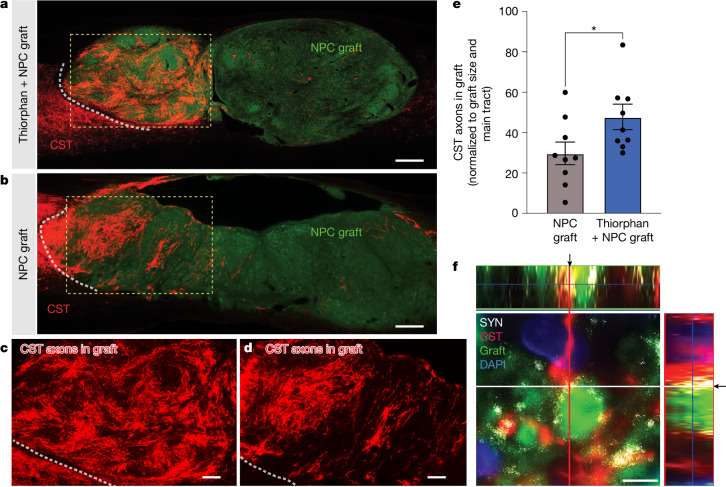
Thiorphan increases corticospinal axonal regeneration. **a**, CST axons labelled anterogradely with Flag (red) show greater regeneration into E14 neural progenitor stem cell grafts (green (green fluorescent protein (GFP))) in animals with thiorphan cortical infusions. The white dotted line indicates the host–graft interface; the boxed region is shown at higher magnification in **c**. **b**, Fewer corticospinal axons regenerate into animals that did not receive thiorphan cortical infusions. The white dotted line indicates the host–graft interface; the boxed region is shown at higher magnification in **d**. **c**,**d**, Higher magnification views of the boxed regions in **a** (**c**) and **b** (**d**) showing regenerating corticospinal axons. **e**, Corticospinal regeneration into graft occupying lesion site is increased 1.6-fold in the presence of thiorphan infusion into the motor cortex (*P < 0.05, two-tailed *t*-test). In the absence of a graft, no host axons are present in the lesion site; non-grafted animals could not be quantified. **f**, Regenerating host corticospinal axons form putative synapses with grafted neurons, based on colocalization of regenerating CST (red) with synaptophysin (SYN, white) apposed to grafted neuron (GFP, green). DAPI in blue. Arrows indicate the exact orthogonal view intersection point, single plane. Scale bars, 500 μm (**a**,**b**); 200 μm (**c**,**d**); 5 μm (**f**). Source data

To examine anatomical mechanisms underlying beneficial effects of thiorphan infusion on functional outcomes, we assessed corticospinal axon regeneration into stem cell grafts occupying the lesion site. Previously, we have shown that corticospinal axons regenerate into NPC grafts placed into sites of SCI and form synapses. Further, grafted NPC neurons extend axons from the lesion site to host neurons below the lesion and form synapses; consequently, new neural relays are formed across the lesion that support functional improvement^13,16–19^. In the present experiment, thiorphan significantly increased corticospinal regeneration into grafts by 60% (*P* < 0.05; Fig. 3a,c,e) compared with individuals that received NPC grafts alone (Fig. 3b,d,e and Supplementary Fig. 3a,b). Thiorphan administration in the absence of NPC grafts could not promote corticospinal regeneration into the lesion cavity because there was no cellular substrate onto which injured corticospinal axons could extend. Thin plane confocal microscopy showed colocalization of corticospinal axon terminals with synaptophysin apposed onto grafted neurons, indicating putative synapse formation from regenerating host axons to grafted neurons (Fig. 3f). Nearly all grafted animals in this experiment exhibited complete graft filling of the lesion cavity (Supplementary Fig. 4a,b). Cortical infusion of thiorphan over 4 weeks caused no damage or neuronal loss in the motor cortex (Supplementary Fig. 4c,d).

We also examined whether thiorphan treatment influenced corticospinal axonal sprouting above the lesion site: there were no significant differences in corticospinal axon density among the four animal groups (Supplementary Fig. 5a,b). Although thiorphan was administered into the motor cortex, we also examined whether there was a difference in the growth of serotonergic or sensory (calcitonin gene-related-peptide-labelled) axons into the graft; as expected, there was no difference between thiorphan + NPC graft and the diluent + NPC grafted groups (Supplementary Fig. 6a–f). We further examined whether thiorphan administration into the cortex influenced the host–graft glial border (glial fibrillary acidic protein immunoreactivity); it did not (Supplementary Fig. 7a–c). Finally, we examined whether thiorphan administration into the cortex affected graft differentiation: it did not (Supplementary Fig. 7d–k); as expected, neither did it affect graft-derived axon extension into the host (Supplementary Fig. 7l,m). Thus, beneficial effects of thiorphan administration were detectable on the corticospinal system to which it was targeted.

### Testing in primate systems

**Fig. 4:**
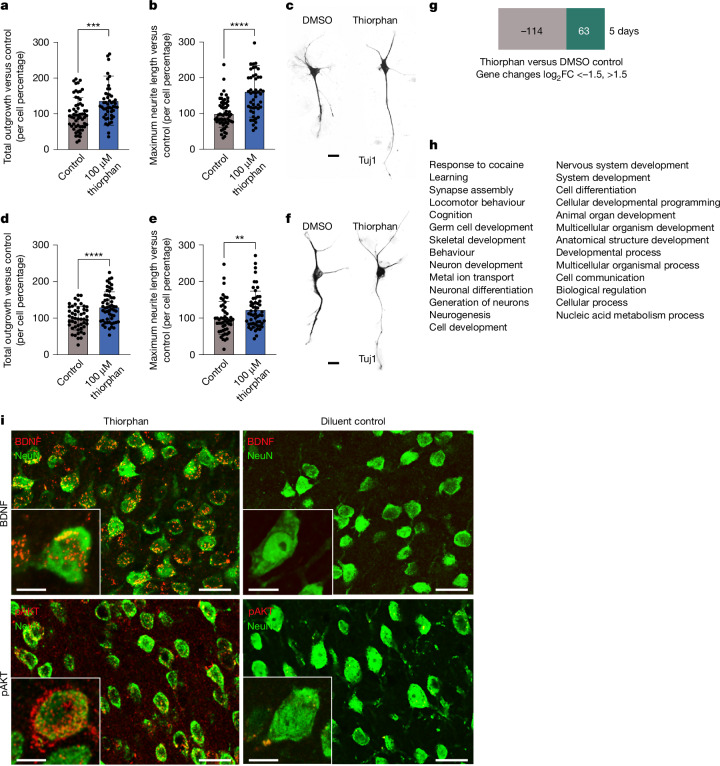
Thiorphan increases growth of monkey and human cortical neurons and induces pro-regenerative state. **a**, Thiorphan significantly increased total neurite outgrowth from primary cultures of adult cynomolgus macaque motor cortex labelled for Tuj (***P < 0.001, two-tailed *t*-test). Control cultures received DMSO. Each point represents the total neurite length of individual neurons. Samples were run as technical duplicates, with neurons cultured for 5 days. **b**, Thiorphan treatment also significantly increased the maximum neurite length per cell by 1.8-fold (****P < 0.0001, two-tailed *t*-test). **c**, Representative images of cultures of adult primate motor cortex treated with diluent or thiorphan. **d**, Thiorphan significantly increased total neurite outgrowth from primary cultures of normal 56-year-old human cortex neurons labelled for Tuj (****P < 0.0001, two-tailed *t*-test). Neurons cultured for 5 days. Each point represents the total neurite length of one neuron. **e**, Thiorphan treatment of human cortical neurons also significantly increased the maximum neurite length per cell (**P < 0.01, two-tailed *t*-test). **f**, Representative images of 56-year-old human cortical neurons in culture labelled for Tuj1. **g**, RNA sequencing was performed on cultures of adult primary motor cortex from rhesus monkeys treated for 5 days with thiorphan or vehicle (DMSO). A total of 177 genes changed ±1.5-fold on a log_2_ scale. **h**, Gene ontology of these 177 genes demonstrates clear enrichment for developmental processes (Gene Ontology database^24^, 10.5281/zenodo.10536401). **i**, To identify in vivo mechanisms recruited by thiorphan treatment, rat brains were removed after 2 weeks of thiorphan infusion and compared with diluent-infused controls. Thiorphan treatment increased immunolabelling for both BDNF and phospho-AKT in the infused motor cortex, reflecting a shift to a state that is developmental and pro-regenerative. Scale bars, 20 μm (**c**,**f**). Scale bars, 25 μm, inset scale bars, 10 μm.

The final step in this process, Step 5, extended the clinical relevance of this work by determining whether thiorphan can also promote growth from adult primate motor cortex and human cortical neurons (Fig. 4a–c). This step is performed last because these samples—particularly normal human cortex samples—are highly valuable. Human participant approval was obtained for these procedures. Neurons were isolated from the monkey M1 motor cortex, dissociated and cultured, adapted from ref. ^2^, and treated with 100 μM thiorphan for 5 days in vitro (two replicates). These cultures are free of NPCs when assessed by markers such as Nestin and Sox2. Thiorphan treatment significantly increased total neurite outgrowth by 36% (*P* < 0.001) and maximum neurite length by 61% (*P* < 0.0001) compared with control conditions (Fig. 4a–c). We then proceeded to culture the human cortex: normal cortex from the middle temporal gyrus of a 56-year-old man was obtained from biopsy, the tissue was dissociated and neuronal cultures established (adapted from ref. ^2^; Supplementary Fig. 8). Treatment with 100 μM thiorphan for 5 days in vitro (Fig. 4d–f) resulted in a significant increase in total neurite outgrowth by 30.3% ± 7.3% (*P* < 0.0001) compared with control conditions. Thiorphan also exhibited a significant increase in longest neurite length by 23.0% ± 9.4% (*P* = 0.016) compared with controls. Thus, thiorphan retains biological activity when tested in monkey and human cortical cultures, exhibiting roughly 40% of the potency exhibited in rats in effects on total neurite outgrowth and 70% of the potency exhibited in rats in terms of longest neurite length.

Thiorphan was identified as a potential candidate for improving axonal growth after CNS injury by virtue of its ability to shift the cellular transcriptome to an embryonic, pro-growth state. To assess whether this mechanism was actually recruited in neuronal target cells, we performed RNA sequencing (RNA-seq) of macaque M1 motor cortex cells exposed to thiorphan for 5 days in vitro compared with control cultures lacking thiorphan exposure (Fig. 4g). Because we had one sample of thiorphan-treated and one sample of vehicle (dimethylsulfoxide (DMSO))-treated cells, we compared mRNA species changing ± 1.5 log_2_ units between the two samples: 177 genes reached this cut-off threshold (Supplementary Fig. 1c). Gene ontology of these 177 genes identified 29 biological processes and remarkably, 16 of these 29 were related to development, including neuron development, nervous system development, and synapse assembly (Fig. 4h). To further examine mechanisms associated with thiorphan delivery, we infused it intracortically for two weeks in rats and performed immunolabelling of the infused region compared with animals that received vehicle infusions: thiorphan administration resulted in increased expression of brain-derived neurotrophic factor (BDNF) (Fig. 4i) and phospho-AKT (Fig. 4i) in the infused region, paralleling higher levels of these molecules observed both in neural development and successful regeneration. These findings support a mechanistic framework in which thiorphan modifies the developmental, metabolic and signalling state of cells to impact neuronal regeneration, consistent with its initial identification on the basis of its ability to replicate the effects of SCI in driving neurons developmentally backward towards a state of embryonic development.

## Discussion

We report the identification of a new and effective drug treatment for SCI arising from an analytical pipeline that targets the regenerative transcriptome of the adult corticospinal motor system. Notably, thiorphan was selected from a database of compounds that have already been tested in human clinical trials, facilitating potential clinical translation. Direct thiorphan infusion into the motor cortex was safe and effective in this experiment, and effects on neurite outgrowth were confirmed in adult rhesus monkey and human cortical cultures. Future studies will determine whether thiorphan exhibits efficacy after intrathecal administration, or after systemic administration because there is transient breakdown of the blood–brain barrier after SCI^20^. In addition, newly synthesized derivatives of thiorphan with improved blood–brain barrier permeability can be developed to simplify administration for clinical trials.

A previous study used in silico screens to identify compounds improving neural repair after peripheral nerve injury, based on dissection of whole DRG including neurons and support cells. The compound ambroxol was identified, which exhibited relatively modest effects in improving peripheral and optic nerve regeneration and cortical sprouting in a model of stroke, without evidence of functional efficacy^6,21^. By contrast, the present study started with a transcriptional dataset obtained from a specific neuronal population that is critical for human voluntary movement after SCI—layer V corticospinal neurons—rather than a general neuronal and glial RNA-seq dataset. We then used a CMap enrichment method^3^ to more deeply probe the CMap dataset that led to the identification of thiorphan, triflusal and milrinone as the most likely potential mimics of the specific corticospinal regeneration transcriptome. We then used a new medium-throughput assay to test candidate drugs in the most faithful potential assay of adult brain neural injury examined to date: cultures of adult motor cortex neurons. Only thiorphan resulted in consistently significant and dose-dependent improvements in motor cortex neurite outgrowth. Other lead candidates (for example, triflusal, milrinone) did not increase neurite outgrowth, suggesting that a rigorous in vitro assay is essential to validate in silico targets, as in silico models may not fully capture the complexity of cellular responses. Subsequent in vivo testing of thiorphan yielded evidence of functional CNS recovery from this type of analytical pipeline. Moreover, thiorphan exhibited significant effects on neurite outgrowth of adult human neurons, supporting clinical translation. This platform can be applied broadly to other CNS disorders, potentially yielding several new candidate drugs to treat CNS injury and neurodegenerative disorders using compounds that have already been tested in humans for other indications and have yielded evidence of safety. A lack of safety results in the failure of up to one-half of human clinical trials^22^, and the current pipeline can de-risk new drug development considerably.

Thiorphan induced recovery of motor function when combined with a neural stem cell graft to a site of severe cervical SCI. This improvement occurred within 2 weeks—a timeframe in which host corticospinal axons have already regenerated into stem cell grafts^1^ and stem cell grafts have extended axons into the host cord below the lesion; indeed, stem cell implants extend axons rapidly into the host beginning 3 days after implantation^16^. Without a stem cell graft in the lesion site, there would be no cellular substrate onto which axons could regenerate into the lesion site with or without thiorphan administration. But in the presence of the stem cell substrate, thiorphan increased corticospinal axon regeneration into the lesion site by 60%. This presumably supported the formation of greater numbers of neural relays across the lesion site^13,16–19^ that, in turn, facilitated functional recovery. The lack of statistically significant recovery in our graft-alone group (*P* = 0.15) highlights the challenge of achieving forelimb functional restoration in this severe model. In previous studies, we used less severe lesion models and found that stem cells alone induced significant functional recovery. Yet in the present more severe lesion, the addition of thiorphan yielded significant and meaningful functional recovery.

Administration of thiorphan in vivo to rats with SCI resulted in elevated levels of BDNF and phospho-AKT in neurons, paralleling higher levels of these molecules observed during neural development. Confirmation that thiorphan actually induces complete reversion of corticospinal neurons to an embryonic transcriptional state^1^ would require RNA-seq studies after in vivo infusions, and these were not performed. RNA sequencing of adult motor cortex cultures from macaque monkeys following thiorphan exposure did indicate a development shift in transcriptional state. Nonetheless, the link between thiorphan treatment and transcriptional change is indirect.

## Conclusions

A pipeline of advanced in silico analysis coupled with improved in vitro screens identify the drug thiorphan as a candidate for the treatment of SCI. This platform can be adopted across nervous system disease states to accelerate the development of new therapies for human neurological disorders.

### Reporting summary

Further information on research design is available in the Nature Portfolio Reporting Summary linked to this article.

## Online content

Any methods, additional references, Nature Portfolio reporting summaries, source data, extended data, supplementary information, acknowledgements, peer review information; details of author contributions and competing interests; and statements of data and code availability are available at 10.1038/s41586-025-09647-y.

## Supplementary information

Supplementary Information Methods, including Supplementary Figs. 1–8.

Reporting Summary

## Source data

Source Data Fig. 2

Source Data Fig. 3

## Data Availability

All code for our analysis can be found at https://github.com/oganm/regenerationCMAP. The RNA sequencing results used in this manuscript can be found at the Gene Expression Omnibus (GSE126957). Source data are provided with this paper.
