# Supplementary material for: Thiorphan reprograms neurons to promote functional recovery after spinal cord injury
Source: Nature. 2025 Oct 29;648(8093):402–8. doi: 10.1038/s41586-025-09647-y (PMC12695623; doi:10.1038/s41586-025-09647-y)
Supplement: Supplementary file 1 — Methods, including Supplementary Figs. 1–8. [file 41586_2025_9647_MOESM1_ESM.pdf]

nature portfolio

https://doi.org/10.1038/s41586-025-09647-y

Supplementary information

Thiorphan reprograms neurons to promote functional recovery after spinal cord injury

In the format provided by the authors and unedited

Supplementary Materials for

Thiorphan Reprograms Neurons to Promote Functional Recovery after SCI

E.A. van Niekerk[1*], C. Marques de Freria[1], B.O. Mancarci[2], K. Groeniger[1], D. Kulinich[1], T. Riley[1], R. Kawaguchi[3], S. Okawa[4], T. Vokes[1], E. Rosenzweig[1], E. Sinopoulou[1], M.J. Castle[1], J.R. Huie[5,6], A.R. Ferguson[5,6], N. Kfoury-Beaumont[7], A. Khalessi[7], P. Pavlidis[2], M.H. Tuszynski[1,8]

*Corresponding author. Email: evanniekerk@health.ucsd.edu; mtuszynski@ucsd.edu

The PDF file includes:

Materials and Methods
Figs. S1 to S8

Materials and Methods

***In Silico* analysis**: The perturbagen (or compound) reference matrix was acquired from the ConnectivityMap R package (Version 1.3, Bioconductor). ConnectivityMap analysis was performed as described in Lamb et al.(*1*) The code for our analysis is described in https://github.com/oganm/regenerationCMAP. The reference matrix included 1309 unique “peturbagens” (small molecules, drugs and compounds). We tested for connectivity of these peturbagens’ profiles in our mouse corticospinal regeneration dataset(*2*) that sampled three time points after induction of regeneration: three days, one week and two weeks. For queries, we selected our differential up- and down-regulated genes from previous published results (GSE126957)(*2*), where regenerating days generated 1320, 791, and 1275 genes respectively across time (FDR ≤ 0.05). These mouse genes were mapped to human orthologs using Homologene(3) resulting in 1252, 742, and 1187 query genes that were also present in the CMAP data, out of a total of 14,516 mouse orthologs present in the CMAP data. These query lists were then used to compute enrichment scores. We limited our analysis to peturbagens that were positively enriched at all three time points (196 peturbagens). “Enrichment connectivity” is a value between +1 and -1, and is a measure of similarity of expression profiles. P-value is calculated by permutation, and the non-null score shows the ratio of experiments that have a consistent score based on the majority of experiments. If the majority has a positive score, it is the ratio of positive scoring experiments to all experiments including those that have 0 or negative scores. “Instance count” is the number of experiments that represent the perturbagen. The specificity and reliability scores were derived from the Connectivity Map web application (https://portals.broadinstitute.org/cmap/, no longer available; accessed May 2017). The specificity score compares the connectivity scores for a perturbagen given the user's query gene list (in our case, genes changing expression during regeneration) to the connectivity scores for the same perturbagen obtained for a set of 312 input gene lists from MSigDB(*4*), with the logic that if a perturbagen is given high connectivity scores for many different query lists, the perturbagen is "non-specific". Specificity is

computed as the proportion of MSigDB gene lists that give a higher connectivity score than the query gene list, which we subtracted from 1.0 so that high scores correspond to higher specificity. The reliability measure is "true" (coded as 1) if the perturbagen is represented by more than one profile in CMap and if the majority of experiments show enrichment in the same direction for the query gene list, and is "false" otherwise (coded as 0). As with the connectivity scores, we took the means of specificity and reliability across the three time points. If the reliability score is false, the result is non-reliable by the standards of the original methodology(*1*). Perturbagens were therefore ranked in order of highest average enrichment score, followed by highest average specificity score across all three time points. If the average reliability score across all three time points were zero, the perturbagen was removed from the ranking. Drug biological annotation was done through the Drug Bank (go.drugbank.com) and an in-depth literature review of top drugs.

Adult mouse primary neuronal cultures: Neurons were isolated from the motor cortex of adult post-natal day 60 C57BL/6 mice, male and female, and cultured as described previously(*5*). Briefly, 3x6 mm blocks of tissue were harvested from the brain region of interest (the motor cortex) and, as solid blocks, placed in an enzymatic buffer solution containing papain and DNase. This tissue was then gently mechanically dissociated for 30 min. This very slow process of dissociation of the starter 3x6 mm tissue block gently teased apart the tissue, permitting for the first time the survival of large numbers of neurons for subsequent culture, as described in(*5*). Debris was then removed using Percoll density gradient centrifugation. For in vitro compound testing, each compound was resuspended in 100% DMSO at a stock concentration of 100mM, including Thiorphan (Bachem, 4010438), Triflusal (Sigma, T6580), Milrinone (Cayman Chem, 25429), and Adiphenine (Sigma, A3649). Compounds were added to culture media at a concentration range of 10 μM to 250 μM within separate respective wells and media was changed every two days for the total culture period of 5 days. Four mouse brains constituted the starting material and were divided into 48 culture wells (Corning, CLS3548-100EA). Three wells were tested per drug concentration, and four concentrations were tested for each drug for a total of 12 wells per drug. For analysis of

mean neurite length per cell and maximal neurite length per cell, data from all measured neurons across three wells were combined. Results were statistically examined using a two-tailed Student's t-test comparing the test condition to the control.

Adult cynomolgus monkey motor cortex primary neuronal cultures: All procedures were carried out in strict adherence to guidelines provided by The Guide for the Care and Use of Laboratory Animals and requirements of the institutional animal welfare committee. Right M1 motor cortex samples were provided from one intact adult male rhesus monkey (age 5.5 years) that is a subject in a separate study examining the transcriptome of the rhesus macaque left motor cortex. The subject was deeply anesthetized with isofluorane, a craniotomy was performed over the right M1 motor cortex, and a 12 mm long x 4 mm wide x 4 mm deep portion of the motor strip was removed over the forelimb region adjacent to the principal sulcus. The brain tissue wet weight was 200mg. The tissue block was then immediately and completely immersed into complete culture media consisting of MACS neuro media (Miltenyi, 130-093-570) supplemented with 10 mg/ml Glutamax (Gibco, 35050061), 10 mg/ml Pen/strep (Sigma, P4333-20ML), 10 mg/ml B27 (Gibco, 17504044), 100 mg/ml FBS (Gibco, 26140) and 0.1 μg/ml BDNF (Peprotech, 450-02). This was transferred on ice to the cell culture facility (15 min). The single block of brain was then divided with a knife into three equally sized pieces measuring 4 mm long x 4 mm wide x 4 mm deep. Brain dissociation was performed as described for mouse cortex above(*5*), with the modification that BDNF (0.02μg/ml) was added to the tissue dissociation buffer that contains papain and DNase. Lab-TekII chamber slides (Nunc, 154534) were coated with poly-L-lysine (Sigma, P4707-50ML) and then, after drying, with 100 μg/mL laminin (Sigma, L2020). Test compounds were added at the concentrations specified above, or the diluent DMSO in controls. Cultures were maintained for five or seven days *in vitro* as previously described(*5*) to fully establish segregated dendritic and axonal compartments(*6*) and quantification of neurite outgrowth was performed as described for mouse cultures.

Adult human cortex primary neuronal cultures: Normal temporal cortex tissue was obtained from a human biopsy specimen under an approved human subjects protocol at UCSD. The tissue consisted of 1 gram of middle temporal gyrus obtained as a 9 mm-long x 4 mm wide x 2 mm thick block of tissue that was divided into three samples each measuring 3 mm x 4 mm x 2 mm. The three blocks of tissue were placed in the operating room into cold MACS neuro media (Miltenyi, 130-093-570) supplemented with 10 mg/ml Glutamax (Gibco, 35050061), 10 mg/ml Pen/strep (Sigma, P4333-20ML), 10 mg/ml B27 (Gibco, 17504044), and 100 mg/ml FBS (Gibco, 26140). The fully submerged blocks were placed on ice and transported to the cell culture lab. Once received in the cell culture lab, the tissue was placed in the same solution as monkey samples listed above; time from initial sample extraction to start of tissue block enzymatic dissociation with papain was 33 minutes. All remaining procedures were as noted above for monkey and mouse tissue. Cells were treated for 5 days *in vitro* with either Thiorphan (100μM) or DMSO (equal volume to Thiorphan) control.

RNA sequencing of cultured monkey adult brain neurons: Monkey adult brain neurons were cultured as described above, with 100μM Thiorphan or DMSO control (volume matched to Thiorphan volume added per well). Cells were cultured in a 48-well plate (Corning, CLS3548-100EA), 500ul volume of media per well. After 5 or 7 days in vitro, RNA was extracted with the "absolutely RNA nanoprep kit" (Agilent, 400753) according to manufacture's instructions. All RNA integrity (RIN) scores were $\geq$ 9.2. Short reads were aligned using STAR to the macaca fascicularis genome. Coverage distribution was estimated using the absolute number of reads aligned where the number of genes with raw aligned read counts between 100 and greater were more than 12,000 for all samples. Uniquely mapped reads were as follows: control 5 days (78.98%), and Thiorphan 5 days (82.76%). EdgR was used for differential expression analysis. Read counts were normalized via trimmed mean method before differential expression analysis. Low expressed genes were removed, genes with Counts Per Million > 0.25 in at least 1 sample were selected for analysis. Gene set enrichment analysis was performed with complete gene list ranked with sign of log Fold Change.

Rat Spinal Cord Surgery: NIH guidelines for laboratory animal care and safety were strictly followed. After undergoing pre-training on the Montoya staircase task, Fischer 344 adult female rats underwent dorsal bilateral C5 severe spinal cord contusions using the Infinite Horizons (IH) Impactor (Precision Systems & Instrumentation, Lexington, KY) with a large impactor probe head of 3.5 mm diameter with 1 s dwell time, applying a force of 200 kilodynes (kdynes) and displacements of 800–1200 µm(*7*). Post-op animals received banamine (2.5–5 mg/kg), ampicillin (3–5 mg/kg) and buprenorphine (Covetrus, 42023-179-05). After post-operative support, animals recovered ambulation but had lasting impairments in forelimb grasping.

Thiorphan Infusion and Neural Stem Cell Grafting: Two weeks after cervical contusion, rats were divided into four groups. **Group 1, Thiorphan** infusion + **NPC graft**: these animals received infusions of Thiorphan into motor cortex for four weeks and implants of spinal cord neural progenitor cells (NPCs) from the E14 spinal cord into the lesion site (N = 9). This group tested the hypothesis that Thiorphan would stimulate corticospinal regeneration into a lesion site containing a substrate permissive for corticospinal regeneration, the NPC graft. Cortex infusions and spinal cord grafting were conducted in the same surgical session. **Group 2, Thiorphan** infusion only (N = 10): these animals received Thiorphan, but lacked a substrate to support corticospinal regeneration into the lesion. It is possible that in these animals, corticospinal sprouting into the spared spinal cord, but not regeneration into the lesion site, could support improved functional outcomes. **Group 3, NPC grafts** only, were a control for the Thiorphan + NPC graft group (N = 9). Previously, in *less* severe models of SCI, NPC grafts have supported recovery of forelimb function(*8, 9*). These animals received infusions of the Thiorphan diluent solution into the motor cortex for four weeks. **Group 4, lesion only**, received neither **Thiorphan** nor **NPC** grafts (N = 9). They received sham injections of stem cell grafting solution into the spinal cord lesion site. A sample size of at least N = 9 per group were determined based on prior studies in similar spinal cord injury models(*8*) demonstrating sufficient power to detect statistically significant differences in forelimb functional recovery and corticospinal axon regeneration following

neural stem cell grafting. All subjects were female and were randomly assigned an experimental group following SCI lesion.

For Thiorphan infusions, lyophilized Thiorphan (Bachem, 4010438) was freshly dissolved in 50% DMSO to a final concentration of 100mM. Diluent controls received infusions of 50% DMSO. 200 μl were loaded into Alzet miniosmotic pumps (Alzet brain infusion kit 3, 0008851). A 30 gauge cannula was inserted into the forelimb motor cortex at stereotaxic coordinates AP 1mm, ML 2mm from bregma on the left cortex and connected to the Alzet minipump, which was placed in the interscapular space. Neural progenitor cells were grafted into the lesion sites as described previously(*8*). Briefly, Fischer 344 E14 spinal cord neural progenitor cells were prepared on the day of grafting from timed pregnant GFP-expressing rat embryos. The spinal cords were dissected and dissociated using papain **(**Miltenyi neural tissue dissociation kit 130-092-628). Cell pellets were resuspended in Fibrinogen (25mg/ml, Sigma, F6755-25MG) containing a 4-factor growth cocktail to support graft survival, modified from Lu et. al.(*10*) containing BDNF (50 ng/μl, Peprotech 452-02), FGF-2 (10 ng/μl, Peprotech 450-33), VEGF (10 ng/μl, Peprotech 400-31) and MDL28170 (50 μM, Sigma M6690). Cells were grafted at a concentration of $9.1 \times 10^5 \pm 0.55$ cells/μl ($\pm$ SD), and placed on ice until engraftment. A total of 3 μl of cells was injected into the contusion site through the dura at a single site using a fine pipette tip and a picoSpritzer II (Parker Hannifin, 052-0500-900).

Functional testing on Montoya staircase: Functional testing assessed skilled forelimb grasping as reported previously(*11*). Briefly, animals were pre-trained on the task for 30 min daily prior to spinal cord contusions, and all animals were required to use the right forelimb for the grasping task. A criterion threshold of 75% retrieval success was used to include animals in the experiment. After contusions, animals were assessed for performance 10 days post-contusion and matched for deficit severity across the four study groups. Then, after Thiorphan infusions and grafting, animals were tested weekly. Outcomes were reported as number of pellets eaten within a 15-minute testing session in the Montoya staircase and accuracy (number of pellets eaten divided by the total number of pellets displaced plus the number of pellets eaten,

multiplied by 100, for the same testing session). All results were scored by an individual blinded to group identity. For statistical analysis of functional outcomes, a generalized estimating equation was used with a Poisson distribution model, and for pellet accuracy a Gamma distribution method was used.

Corticospinal tract anterograde tracing: 12 weeks after SCI, corticospinal axons were anterogradely traced by injections into the motor cortex of an AAV9 vector expressing a FLAG tag, AAV9-CAG-Ruby2sm_FLAG, as previously described(*8*). Two weeks later, subjects were transcardially perfused with 4% paraformaldehyde.

Tissue processing: Brains were removed and sectioned on a microtome set at 35 µm intervals in the coronal plane to assess cortical infusion sites. Spinal cords were sectioned on a cryostat set at 35 µm intervals in the sagittal plane to assess axonal regeneration. The antibodies used for immunolabeling were anti-FlagM2 (D6W5B rabbit monoclonal at 1:1000, Cell Signaling, 14793), anti-GFP (goat polyclonal at 1:1000, Rockland, 600-101-215) and synaptophysin (mouse monoclonal at 1:1000, GeneTex, GTX633821). For immunolabeling of cells in culture, cells were fixed with 8% PFA by removing only half of the culture media, and adding an equal volume of 8% PFA for a final concentration of 4% PFA, incubated at room temp for 20 min. PFA media was then gently removed, and washed once with 1XPBS for 30 min standing stationary. PBS was gently removed and cells blocked and permeabilized in 5% donkey serum in 0.25% TritonX100 for 1 hour at room temp. Permeabilization buffer was removed and cells incubated in Tuj1 primary antibody (at 1:5000, Biolegend, 802001) overnight at 4°C standing stationary, followed by 568-donkey anti rabbit secondary antibody (at 1:1000, Jackson Immuno, 711-065-152) dissolved in 5% donkey serum for 1hr standing stationary. Tuj1 antibody was pre-blocked for 24hrs by placing antibody in 5% donkey serum at 1:5000 and rotating overnight at 4°C to remove any non-specific labeling, and then added to fixed cells.

Imaging and histological quantification: Images of brain or spinal cord sections were captured using a Keyance BZ-X710 microscope (Version 1.3.1.1, Nikon CFI 60 series).

For quantification of axon regeneration in spinal cord sections, each image was captured and total pixel number representing Flag-labeled axons was quantified. The total graft area was defined using GFP labeling. A total of 3 sections per animal were quantified in this manner, and total axon pixels were divided by total graft area sampled per subject. To control for potential differences in tracing efficiency between animals, this value per animal was divided by the density of FLAG-labeled axons in the main corticospinal tract in the spinal cord at the C2 levels. All assessment and quantification were performed by one individual blinded to group identity.

Assessment of Thiorphan activity in motor cortex - Neprilysin assay: To assess whether Thiorphan remained active over the four-week infusion period into the motor cortex, additional animals underwent 1, 2 or 4 week infusions of Thiorphan into the motor cortex using Alzet minipumps as described above. At the end of this time period, the motor cortex was homogenized and tested for its ability to cleave a synthetic neprilysin substrate peptide. Once cleaved, the fluorometric product (Ex/Em=330/430 nm) was measured. Samples included animals that received infusions of the diluent, DMSO, N = one animal per group per time point, three technical replicates per condition. Activity levels were measured in the infused (left) motor cortex, the non-infused right motor cortex, and in a control region, the cerebellum. The assay was sensitive to 20 μmol/mg tissue. Results were expressed in relative fluorescence units (RFU Ex/Em = 330/340 nm). Results of three technical replicates per condition were compared using unpaired Student's two-tailed t-test.

Statistics – Statistical analyses were performed by a statistical core (RH, ARF). A generalized linear model (GlzM) with gamma probability distribution was used to test for group differences on percentage data where appropriate; GlzM is robust to non-normal distribution. Posthoc univariate generalized linear model analyses of percent change from controls used a bonferoni correction to adjust for multiple comparisons. A generalized estimating equation using a Poisson distribution was used to test for differences in behavioral count data between groups over time, and gamma distribution for percentage accuracy data. A robust estimator was used for the covariance matrix,

and an autoregressive working correlation matrix was specified, which assumes that for each subject, the measure at a particular timepoint is correlated with measure from the previous and next time point. This approach is an extension of the generalized linear model, and is more robust to missing data and non-normal distribution that other repeated measures statistical tests (such as repeated measures analysis of variance). Statistical analyses were performed using SPSS v29.0.

References

1. J. Lamb *et al.*, The Connectivity Map: using gene-expression signatures to connect small molecules, genes, and disease. *Science* **313**, 1929-1935 (2006).
2. G. H. D. Poplawski *et al.*, Injured adult neurons regress to an embryonic transcriptional growth state. *Nature* **581**, 77-82 (2020).
3. D. L. Wheeler *et al.*, Database resources of the National Center for Biotechnology Information. *Nucleic Acids Res* **36**, D13-21 (2008).
4. A. Liberzon *et al.*, Molecular signatures database (MSigDB) 3.0. *Bioinformatics* **27**, 1739-1740 (2011).
5. E. A. van Niekerk *et al.*, Methods for culturing adult CNS neurons reveal a CNS conditioning effect. *Cell Rep Methods* **2**, 100255 (2022).
6. C. G. Dotti, C. A. Sullivan, G. A. Banker, The establishment of polarity by hippocampal neurons in culture. *J Neurosci* **8**, 1454-1468 (1988).
7. C. M. Freria, L. Graham, A. Azimi, P. Lu, Adaptation of a cervical bilateral contusive spinal cord injury for study of skilled forelimb function. *Exp Neurol* **360**, 114275 (2023).
8. K. Kadoya *et al.*, Spinal cord reconstitution with homologous neural grafts enables robust corticospinal regeneration. *Nat Med* **22**, 479-487 (2016).
9. J. H. Brock, L. Graham, E. Staufenberg, S. Im, M. H. Tuszynski, Rodent Neural Progenitor Cells Support Functional Recovery after Cervical Spinal Cord Contusion. *J Neurotrauma* **35**, 1069-1078 (2018).
10. P. Lu *et al.*, Long-distance growth and connectivity of neural stem cells after severe spinal cord injury. *Cell* **150**, 1264-1273 (2012).
11. C. P. Montoya, L. J. Campbell-Hope, K. D. Pemberton, S. B. Dunnett, The "staircase test": a measure of independent forelimb reaching and grasping abilities in rats. *J Neurosci Methods* **36**, 219-228 (1991).

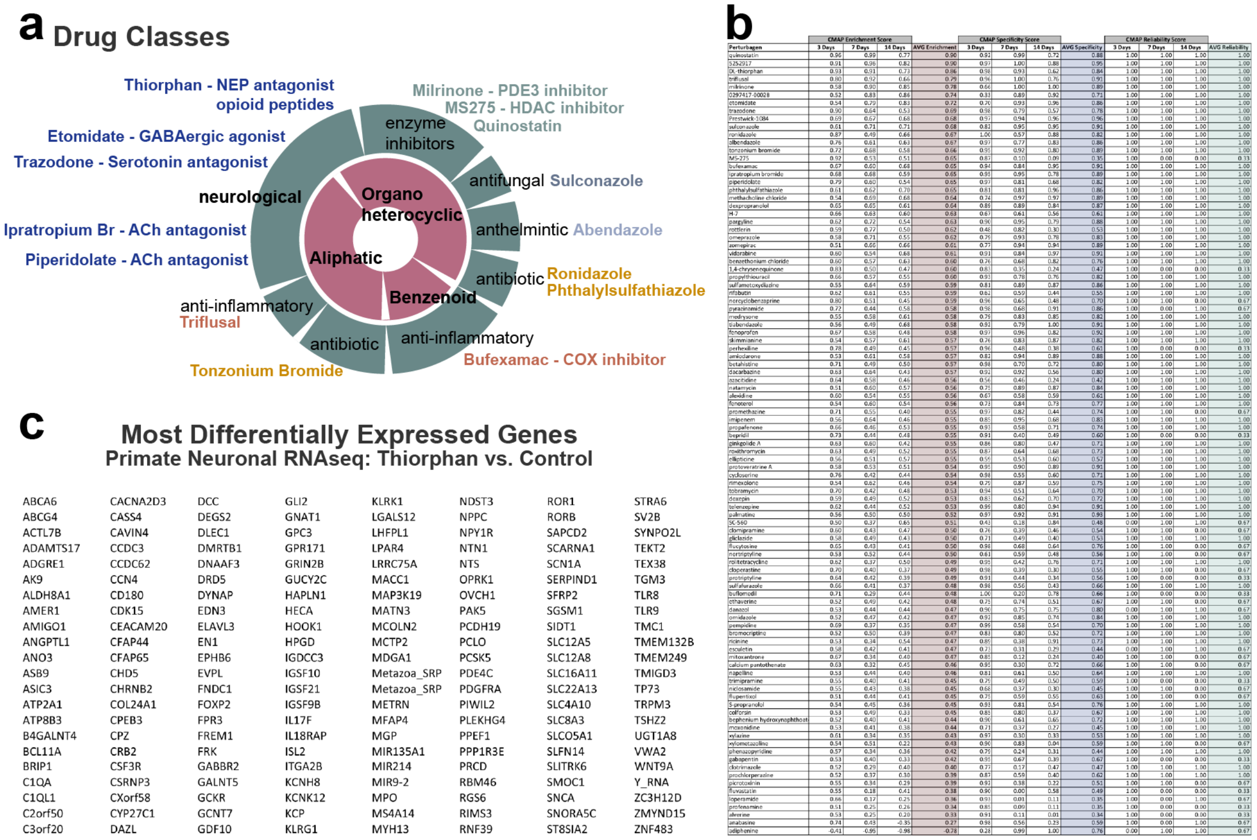

Fig. S1. (**A**) Drug classes of top 15 “hits” from *in silico* analysis classified by CMAP, excluding 5252917, 0297417-0002B, and Prestwick-1084 because no information is available for these compounds. (**B**) CMAP analysis top drugs shown where the connectivity score, reliability score, and inverted specificity score is listed for each time point after regeneration. (**C**) RNA sequencing of macaque M1 motor cortex cells exposed to Thiorphan for 5 days *in vitro* compared to control cultures lacking Thiorphan exposure identified 177 mRNA species changing ± 1.5 $\log_2$ units between the two samples. Here we list all 177 mRNAs.

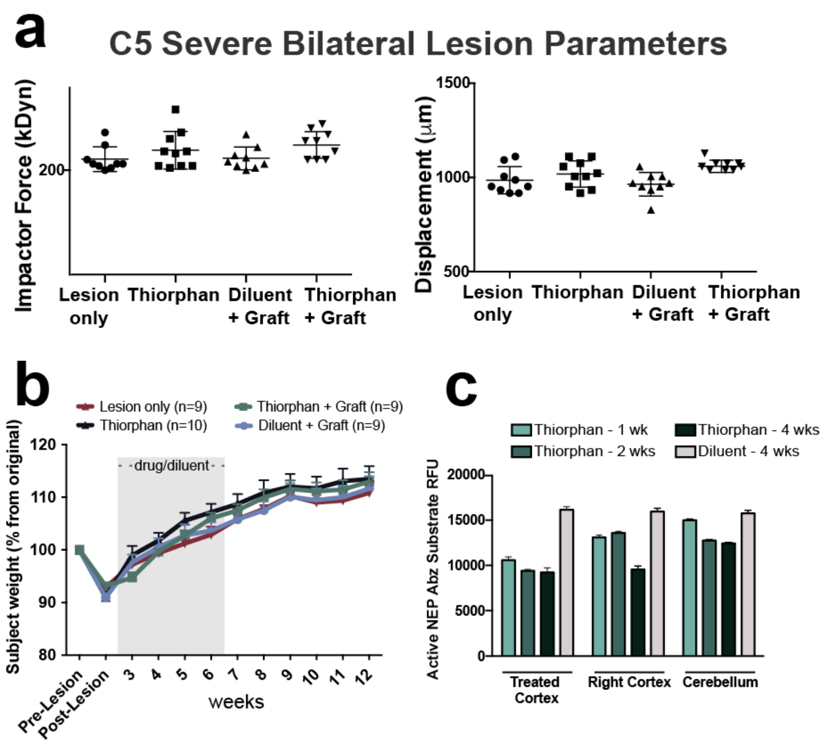

Fig. S2. (**A**) C5 bilateral contusion lesion parameters: there were no significant differences in measured impact parameters among groups. Device set to impactor force 200 kDyn and displacement 1.0 mm. (**B**) Infusions were well-tolerated, as evidenced by equivalent weight gain in all groups over time. (**C**) Neprilysin activity assay of brain tissue treated with Thiorphan or DMSO control for 1, 2 or 4 weeks. Absorbance RFU at 340 nm. Error bars ± SEM of three technical replicates per condition.

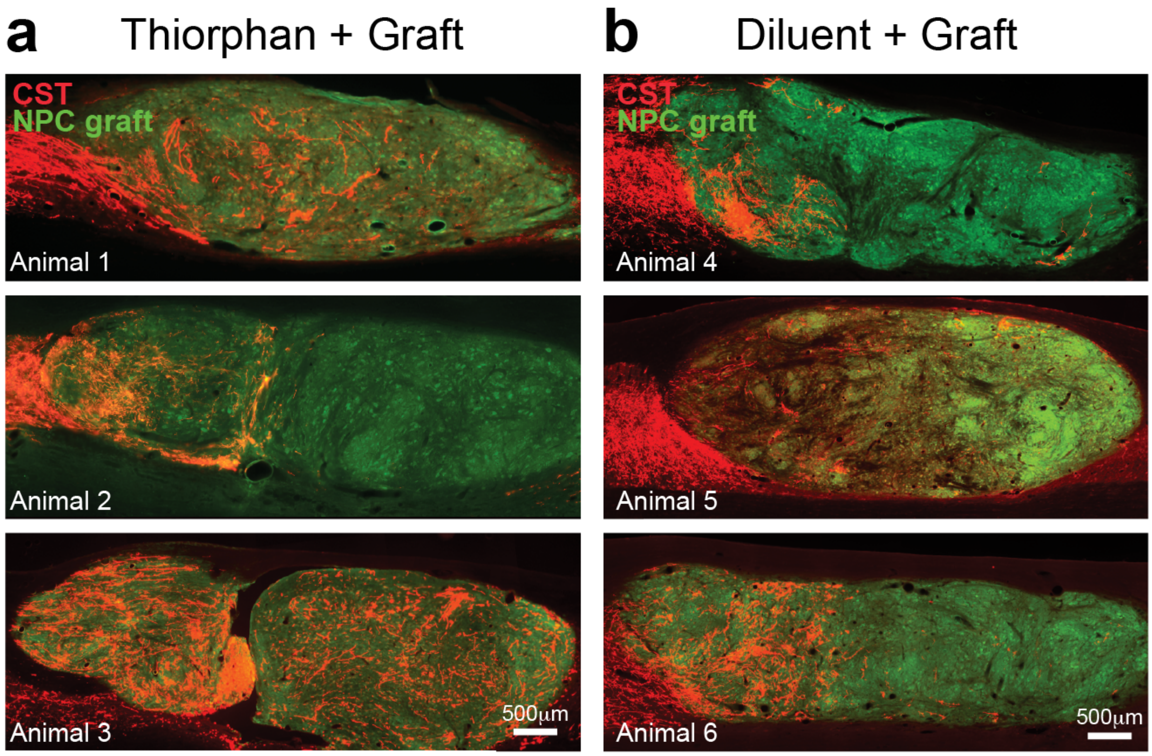

Fig. S3. Corticospinal Tract Regeneration
(**A**) Examples of corticospinal tract (CST) regeneration into NPC grafts in three different animals treated with Thiorphan, three months after severe C5 bilateral contusion. Scale bar 500 µm. **(B)** Corticospinal regeneration in three different animals treated with diluent control. Scale bar 500 µm.

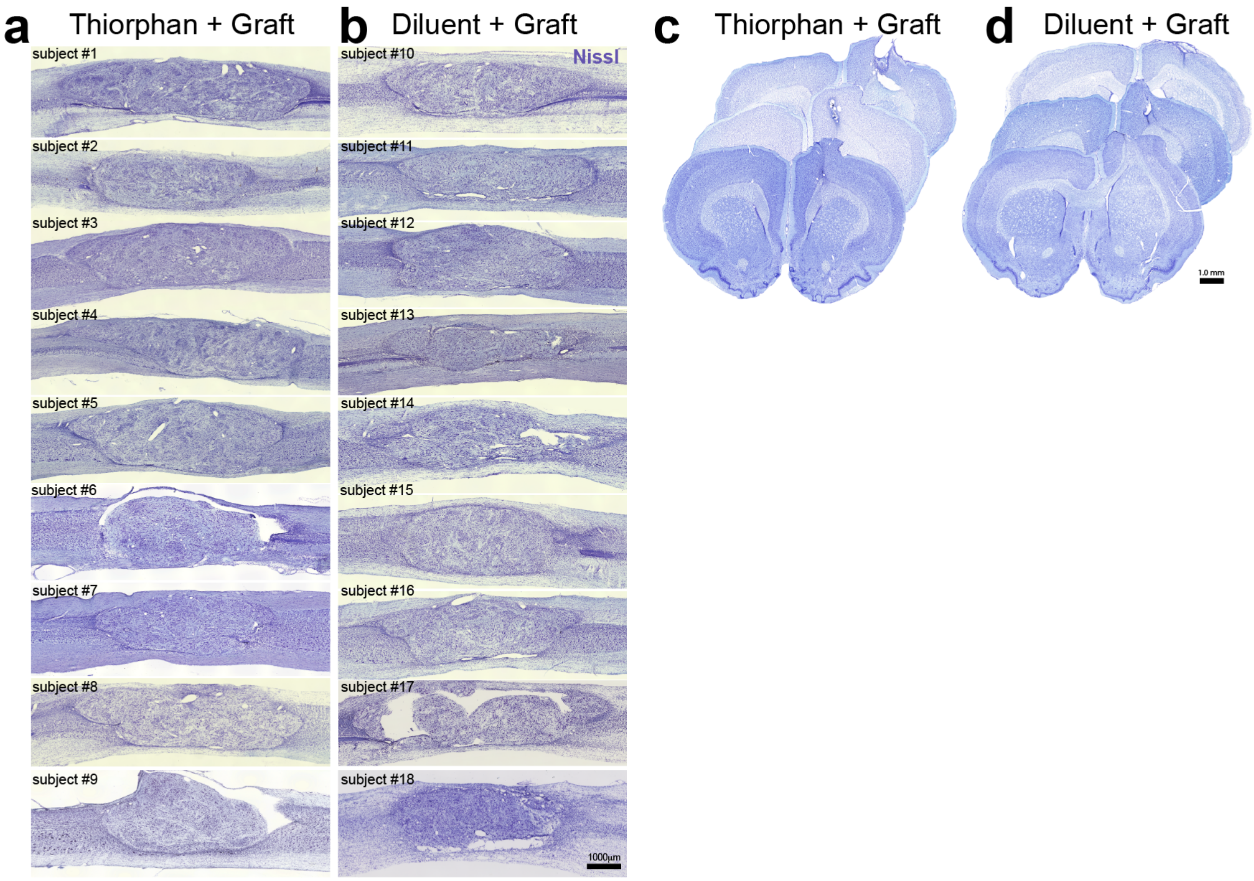

Fig S4. (**A-B**) Nissl stains of sagittal sections containing grafts in all animals (Keyance, 10X objective image acquisition). Rostral is left. Scale bar 1.0 mm. (**C**) Representative images of cortical infusion sites in animals that received 4 weeks of either Thiorphan or (**D**) diluent control. Coronal sections. 10X objective image acquisition. Scale bar 1.0 mm.

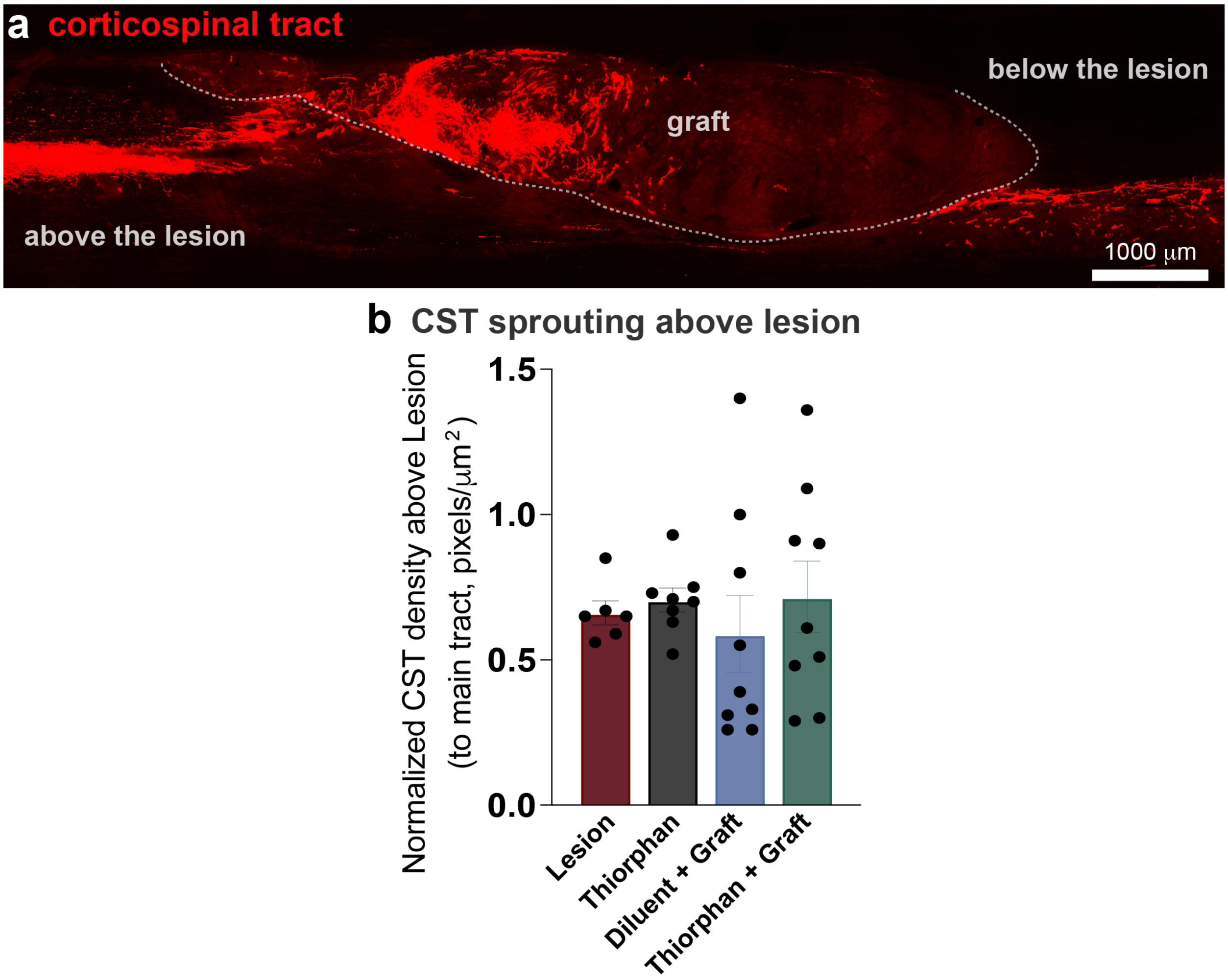

Fig. S5. Corticospinal Tract Growth
(**A**) Overview of horizontal section of rat corticospinal tract; rostral is left and caudal right. Axonal regeneration into graft (dashed line) is evident. Scale bar 1mm. **(B)** Corticospinal sprouting above the lesion site was quantified within a 1mm block rostral to the lesion, and values were normalized to axonal density in the main tract. There is no significant difference between treatment groups. Each data point represents one animal. $\pm$ SEM.

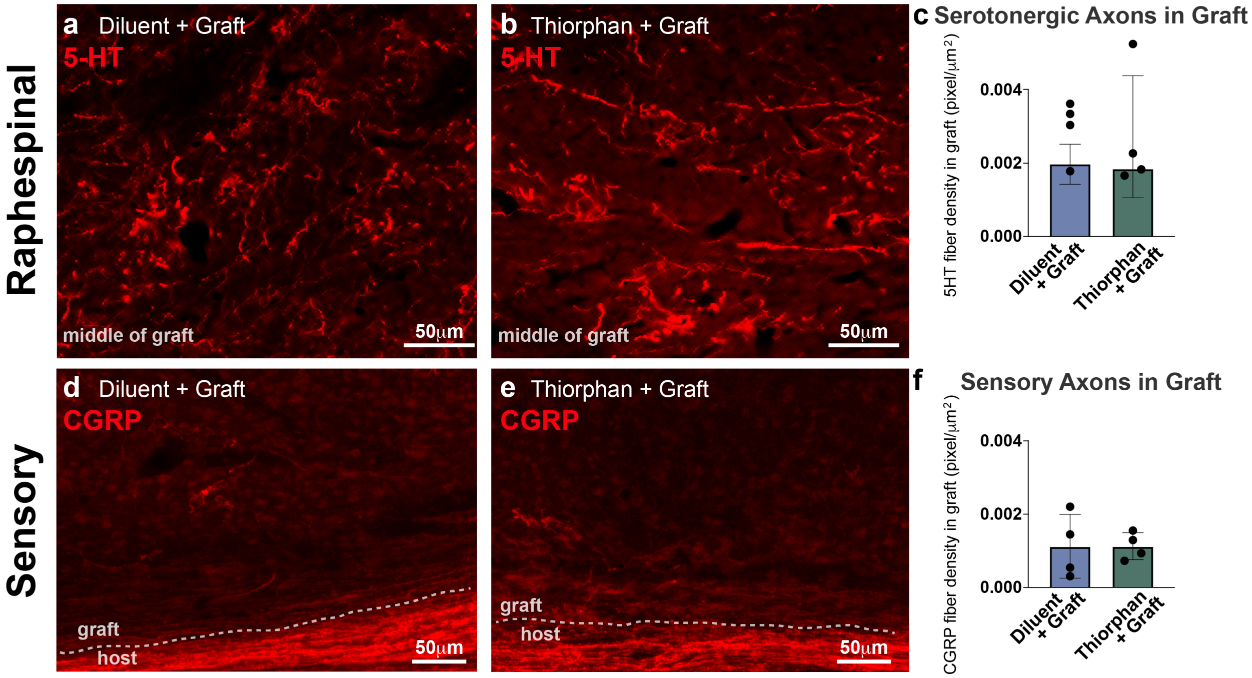

Fig. S6. Assessment of Serotonergic and Sensory Axon Growth into Grafts
(**A-C**) 5HT-labeled raphespinal axons were quantified within the graft among Thiorphan- and Diluent-treated groups. There were no significant differences (P = 0.84).
(**D-F**) Similarly, there were no significant differences between groups in growth of sensory, CGRP-labeled axons into grafts (P = 0.99). Each data point represents one animal $\pm$ SEM. Scale bars 50 μm.

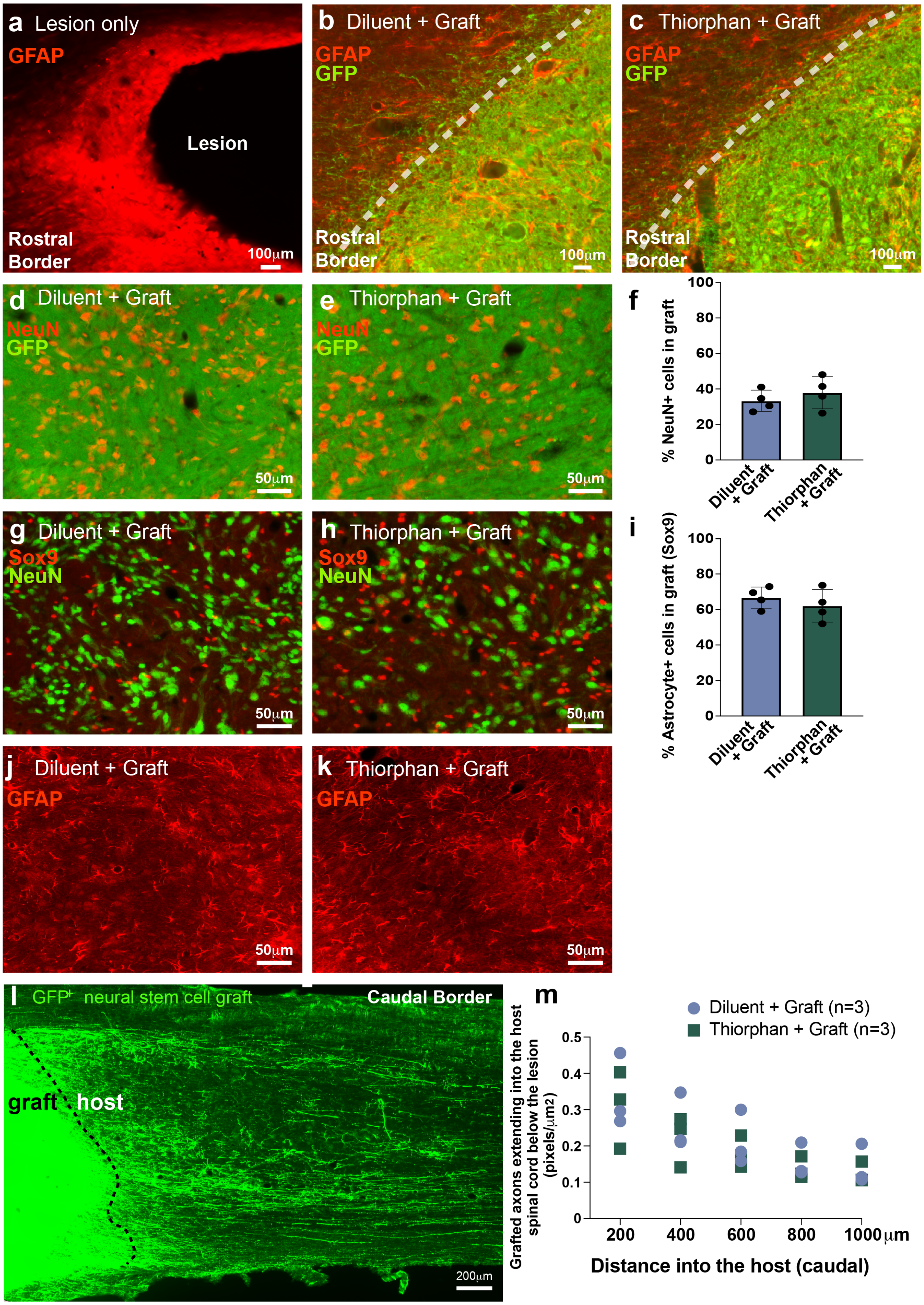

a Lesion only
GFAP
Lesion
Rostral Border
100µm
b Diluent + Graft
GFAP
GFP
Rostral Border
100µm
c Thiorphan + Graft
GFAP
GFP
Rostral Border
100µm
d Diluent + Graft
NeuN
GFP
50µm
e Thiorphan + Graft
NeuN
GFP
50µm
f
% NeuN+ cells in graft
Diluent + Graft
Thiorphan + Graft
g Diluent + Graft
Sox9
NeuN
50µm
h Thiorphan + Graft
Sox9
NeuN
50µm
i
% Astrocyte+ cells in graft (Sox9)
Diluent + Graft
Thiorphan + Graft
j Diluent + Graft
GFAP
50µm
k Thiorphan + Graft
GFAP
50µm
l GFP+ neural stem cell graft
Caudal Border
graft
host
200µm
m
Diluent + Graft (n=3)
Thiorphan + Graft (n=3)
Grafted axons extending into the host spinal cord below the lesion (pixels/µm2)
Distance into the host (caudal)
1000µm

Fig. S7. (A) GFAP labeling to identify host astrocyte responses to lesion alone. There is an increase in GFAP labeling surrounding the lesion site in animals with lesions and no grafts. **(B-C)** The presence of a graft attenuates the formation of a glial border at the host-graft interface. The extent of glial border attenuation does not differ between the two grafted groups, indicating that Thiorphan did not improve axon regeneration by attenuating the glial border. Scale bar 100 μm. **(D-K)** Treatment with Thiorphan also did not affect graft differentiation, as measured by the proportion of cells in the graft that express (**D-K**) NeuN, (**G-I**) Sox9 (for astrocytes), or (**J-K**) GFAP (for astrocytes). The percentage of NeuN and Sox9 cells was calculated relative to total DAPI-labeled cells. Scale bars **A-K**, 50 μm. $\pm$ SEM (**L-M**) Treatment with Thiorphan did not influence axon emergence from grafted neural stem cells into the host spinal cord caudal to the lesion. Scale bar 200 μm

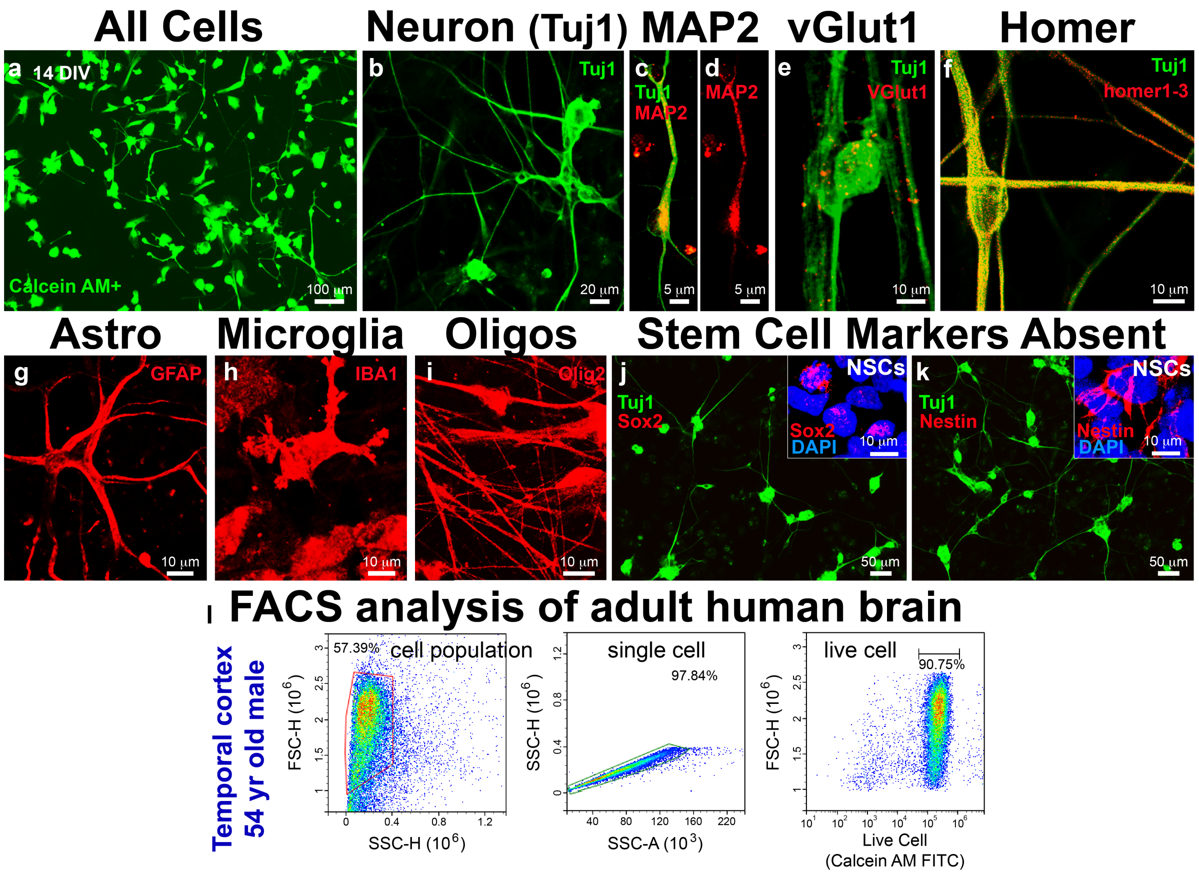

Fig. S8. (**A**) Medium magnification view of neuronal cultures from adult human cortex biopsy, 14 days in culture. Calcein AM+ assay for live cells. Scale bar 100 μm. **(B)** Neurons express the neuron-specific marker **Tuj1**. Scale bar 20 μm. **(C-D)** Neurons also express **MAP2** in the somatodendritic compartment and Tuj1 additionally in the axon, consistent with normal labeling of neurons for this marker. Scale bar 5 μm. **(E)** **VGlut1** (vesicular glutamate transporter) identifies excitatory synaptic vesicles with Tuj1 in green, scale bar 10 μm. **(F)** **Homer1-3** associates with post-synaptic densities localized along Tuj1-labeled processes. Scale bar 10μm. **(G)** **GFAP** labeling of astrocytes. Scale bar 10 μm. **(H)** **IBA1** labeling identifies microglia. Scale bar 10 μm. **(I)** **Olig2** labeling for oligodendrocytes. Scale bar 10 μm. **(J, K)** Labeling for neural progenitor cell markers **Sox2**and **Nestin** in adult human brain cultures did not detect cells labeled for these markers. Scale bar 50μm. Inset shows positive control labeling for Sox2 and Nestin, respectively, in separate neural stem cell (NSC) cultures. Scale bar 10μm. **(L)** FACS analysis from 54 y.o. human cortex indicates the presence of 10,500 neurons. This estimate is derived from flow cytometry, where we recorded the number of detected events within a known sample volume. Applying a P1 gating strategy to exclude debris (left graph), 57.4% of recorded events correspond to intact cells, providing a total number of 58,000 cells that are in single cell suspension (middle graph, 97%). Of these single cells, viability assessed with Calcein AM+ assay (right graph) demonstrates that 90.8% of cells are viable after dissociation and establishment of the cultures. Because the human cortex is comprised of ~20% neurons, the total number of neurons isolated is estimated at 10,500.
