# Supplementary material for: Thiorphan reprograms neurons to promote functional recovery after spinal cord injury
Source: Nature. 2025 Oct 29;648(8093):402–8. doi: 10.1038/s41586-025-09647-y (PMC12695623; doi:10.1038/s41586-025-09647-y)
Supplement: Supplementary file 2 — Reporting Summary [file 41586_2025_9647_MOESM2_ESM.pdf]

nature portfolio

Corresponding author(s): Erna van Niekerk, Mark Tuszynski

Last updated by author(s): Jul 17, 2024

Reporting Summary

Nature Portfolio wishes to improve the reproducibility of the work that we publish. This form provides structure for consistency and transparency in reporting. For further information on Nature Portfolio policies, see our Editorial Policies and the Editorial Policy Checklist.

Statistics

For all statistical analyses, confirm that the following items are present in the figure legend, table legend, main text, or Methods section.

n/a	Confirmed	
☐	☒	The exact sample size (*n*) for each experimental group/condition, given as a discrete number and unit of measurement
☐	☒	A statement on whether measurements were taken from distinct samples or whether the same sample was measured repeatedly
☐	☒	The statistical test(s) used AND whether they are one- or two-sided<br>*Only common tests should be described solely by name; describe more complex techniques in the Methods section.*
☐	☒	A description of all covariates tested
☐	☒	A description of any assumptions or corrections, such as tests of normality and adjustment for multiple comparisons
☐	☒	A full description of the statistical parameters including central tendency (e.g. means) or other basic estimates (e.g. regression coefficient) AND variation (e.g. standard deviation) or associated estimates of uncertainty (e.g. confidence intervals)
☐	☒	For null hypothesis testing, the test statistic (e.g. *F*, *t*, *r*) with confidence intervals, effect sizes, degrees of freedom and *P* value noted<br>*Give P values as exact values whenever suitable.*
☐	☐	For Bayesian analysis, information on the choice of priors and Markov chain Monte Carlo settings
☐	☐	For hierarchical and complex designs, identification of the appropriate level for tests and full reporting of outcomes
☐	☐	Estimates of effect sizes (e.g. Cohen's *d*, Pearson's *r*), indicating how they were calculated

Our web collection on statistics for biologists contains articles on many of the points above.

Software and code

Policy information about availability of computer code

Data collection	The RNA sequencing data used in this manuscript can be found at the Gene Expression Omnibus (GSE126957).
Data analysis	In this study, we utilized open source code from R, version 3.4.3. The code empolyed is known as Connectivity Map (https://github.com/PavlidisLab/regenerationCMap) which has been previously published and is freely accessible as open source code (Lamb J. et. al., Science 2006 313:1929-35). No custom or commercial code was used in the data analysis.

For manuscripts utilizing custom algorithms or software that are central to the research but not yet described in published literature, software must be made available to editors and reviewers. We strongly encourage code deposition in a community repository (e.g. GitHub). See the Nature Portfolio guidelines for submitting code & software for further information.

Data

Policy information about availability of data

All manuscripts must include a data availability statement. This statement should provide the following information, where applicable:

- Accession codes, unique identifiers, or web links for publicly available datasets
- A description of any restrictions on data availability
- For clinical datasets or third party data, please ensure that the statement adheres to our policy

The RNA sequencing data used in this manuscript can be found at the Gene Expression Omnibus (GSE126957).

Research involving human participants, their data, or biological material

Policy information about studies with human participants or human data. See also policy information about sex, gender (identity/presentation), and sexual orientation and race, ethnicity and racism.

Reporting on sex and gender	*Use the terms sex (biological attribute) and gender (shaped by social and cultural circumstances) carefully in order to avoid confusing both terms. Indicate if findings apply to only one sex or gender; describe whether sex and gender were considered in study design; whether sex and/or gender was determined based on self-reporting or assigned and methods used. Provide in the source data disaggregated sex and gender data, where this information has been collected, and if consent has been obtained for sharing of individual-level data; provide overall numbers in this Reporting Summary. Please state if this information has not been collected. Report sex- and gender-based analyses where performed, justify reasons for lack of sex- and gender-based analysis.*
Reporting on race, ethnicity, or other socially relevant groupings	*Please specify the socially constructed or socially relevant categorization variable(s) used in your manuscript and explain why they were used. Please note that such variables should not be used as proxies for other socially constructed/relevant variables (for example, race or ethnicity should not be used as a proxy for socioeconomic status). Provide clear definitions of the relevant terms used, how they were provided (by the participants/respondents, the researchers, or third parties), and the method(s) used to classify people into the different categories (e.g. self-report, census or administrative data, social media data, etc.) Please provide details about how you controlled for confounding variables in your analyses.*
Population characteristics	*Describe the covariate-relevant population characteristics of the human research participants (e.g. age, genotypic information, past and current diagnosis and treatment categories). If you filled out the behavioural & social sciences study design questions and have nothing to add here, write "See above."*
Recruitment	*Describe how participants were recruited. Outline any potential self-selection bias or other biases that may be present and how these are likely to impact results.*
Ethics oversight	The UCSD Institutional Review Board Administration approved the protocol (IRB# 801790, version 17)

Note that full information on the approval of the study protocol must also be provided in the manuscript.

Field-specific reporting

Please select the one below that is the best fit for your research. If you are not sure, read the appropriate sections before making your selection.

☒ Life sciences ☐ Behavioural & social sciences ☐ Ecological, evolutionary & environmental sciences

For a reference copy of the document with all sections, see nature.com/documents/nr-reporting-summary-flat.pdf

Life sciences study design

All studies must disclose on these points even when the disclosure is negative.

Sample size	N=1
Data exclusions	none
Replication	none
Randomization	none
Blinding	none

Reporting for specific materials, systems and methods

We require information from authors about some types of materials, experimental systems and methods used in many studies. Here, indicate whether each material, system or method listed is relevant to your study. If you are not sure if a list item applies to your research, read the appropriate section before selecting a response.

Materials & experimental systems

n/a	Involved in the study
☐	☒ Antibodies
☐	☐ Eukaryotic cell lines
☐	☐ Palaeontology and archaeology
☐	☒ Animals and other organisms
☐	☐ Clinical data
☐	☐ Dual use research of concern
☐	☐ Plants

Methods

n/a	Involved in the study
☐	☐ ChIP-seq
☐	☐ Flow cytometry
☐	☐ MRI-based neuroimaging

Antibodies

Antibodies used

REAGENT or RESOURCE SOURCE IDENTIFIER
Antibodies
Synaptophysin GeneTex GTX633821 lot# 42667
DYKDDDDK Tag (D6W5B) Anti-FlagM2 Cell Signalling 14793S lot# 7
Anti-GFP Rockland 600-101-215 lot# 35577
NeuN Encore MCA-1B7 lot# 091918
Biotin-SP -conjugated AffiniPure Donkey Anti-Mouse IgG (H+L) Jackson ImmunoResearch 715-065-150 lot# 132471
Biotin-SP-conjugated AffiniPure Donkey Anti-Rabbit IgG (H+L) Jackson ImmunoResearch 711-065-152 lot# 165233
AlexaFlour™ 647 Donkey Anti-Mouse IgG (H+L) Invitrogen A31571 lot# 2555690
AlexaFluor™ 488 Donkey Anti-Goat IgG (H+L) Invitrogen A11055 lot# 2604365
Streptavidin AlexaFluor™ 568 conjugate Invitrogen S11226 lot# 2397941
Chemicals, peptides, and recombinant proteins
Ketamine Covetrus 11695-0703-1 Lot# 20022096
Xylazine Covetrus 11695-4024-1 lot# 2D012
Acepromazine Maleate Covetrus 11695-0095-5 lot# 20612930
Banamine Covetrus 11695-4025-1 Lot# 2C036
Buprenorphine Hydrochloride Covetrus 42023-179-05 Lot# 59009
Meloxicam Covetrus 11695-6936-2 Lot# 2471-90D
Ampicillin Covetrus 55150-113-10 Lot# AP0123006A
Lactated Ringers Solution Vetivex 17033-491-25 Lot# 2302101
paraformaldehyde Fisher Scientific P0018500G lot# URRTC-OU
1XPBS Gibco 14190-144 lot#2290385
BDNF Peprotech 450-02
MACS neuro media Miltenyi 130-093-570
10% BSA sterile stock solution Miltenyi 130-91-376
B27 Gibco 17504044
100X Glutamax Gibco 35050061
100X Pen/strep Sigma P4333-20ML
FBS Gibco 26140
70-µM cell strainer Falcon 352350
0.01% poly-L-lysine Sigma P4707-50ML
Laminin Sigma L2020
Fibrinogen Sigma F6755-25MG
FGF-2 Peprotech 450-33
VegF Peprotech 400-31
MDL28170 Sigma M6690
Double distilled H2O Invitrogen 10-977-023
Nunc Lab-Tek II 8-well chambered slide Sigma Z734853-96EA
48 culture well plate Corning CLS3548-100EA
DPBS with Ca2+ and Mg2+, glucose, pyruvate Gibco 14287072
DPBS with Ca2+ and Mg2+ Gibco 14040141
Isoflurane gas Covetrus 029405
Thiorphan Bachem 4010438
Triflusal Sigma T6580
Milrinone Cayman Chem 25429
Adephinine Sigma A3649
DMSO Sigma D2650
Tissue Plus O.C.T. Compound Scigen 4586 lot# 4694
Sucrose Fisher Chemical S5-500 lot# 223110
Ethylene glycol Fisher Chemical E178-4 lot# 224188
Glycerol Fisher Chemical G33-4
Triton-X100 Fisher Scientific BP151-100 lot#158296
Sodium Borohydride (NaBH4) SigmaAldrich 452882-100G lot# 27796CPV
Sudan Black B Fisher Chemical C29H24N6 lot# 164021
30% H2O2 Fisher Chemical H325-500 lot# 223427
Fluoromount-G® Southern Biotech 0100-01 lot# K0322-Y332
chloroform HPLC grade Fisher Chemical C606-1 lot# 223733
200 proof ethanol Koptec 64-17-5 lot# A09142301D

2-propanol HPLC grade Fisher Chemical A451-4 lot# 223462
thionin acetate Beantown Chemical 215525-25G lot# 50001565
Citrisolv clearing agent Decon Laboratories 5989-27-5 lot# 220772
DPX Mounting media Sigma Aldrich 06522-500ML lot# 102653547
gelatin type A Sigma G2500-1KG lot# SLBV5185
tyramine hydrochloride Sigma T2879-1G
succinimidyl-6-(biotinamido) hexanoate Santa Cruz Biotech sc-215916
VECTASTAIN® Elite® ABC Kit, Peroxidase (Standard) Vector Laboratories PK-6100 lot# ZK0316
DAB Substrate Kit, Peroxidase (With Nickel) Vector Laboratories SK-4100 lot# ZJ1215
Superfrost Plus™ Microscope Slides Fisherbrand 1255015 lot# 23915
VWR® Coverslips Fisher Scientific 48393-081 lot# 23307
Critical commercial assays
Adult brain dissociation kit Miltenyi 130-107-677
Neuron isolation kit Miltenyi 130-115-390
Nugen Ovation RNA Ultra Low V2 Nugen 0344NB
Absolutely RNA nanoprep kit Agilent 400753
Deposited data

Other
Brain Infusion Kit Alzet 0008851
Mini Osmotic Pump Alzet 2004
Glass Capillaries World Precision Instruments 1B150F-4
Picospritzer II Parker Hannifin 052-0500-900
Infinite Horizons Impactor Precision Systems & Instrumentation IH-0400
Masterflex Console Drive for perfusions Cole and Parmer Instrument Company 77202-60
SteriFlip Vacuum Filtration System Miltenyi SCGP00525
Montoya Staircase (Rat) Lafayette Instruments 80300A
Sliding Microtome Thermo Scientific HM 450
Cryostat Leica 3050 S
Supreme Mini-Treats™, Certified Bio-Serv F05472-1
Dustless Precision Pellets®, Sucrose Bio-Serv F0023
Teklad Rodent Diet Envigo NC1434463
Diet Gel Recovery® ClearH20 72-06-5022
Hydrogel® ClearH20 70-01-5022
Diamond Dry Cellulose Bedding Tecklad 7070C
Software and algorithms
NovoExpress software Agilent Version 1.5.0
ConnectivityMap R package Bioconductor Version 1.3
Fiji (Image J) Image J Version 1.4.1
Imaris software for 3D rendering Imaris Version 9.5
ANY-maze tracking software ANY-maze Version 7.0

Validation

per manufacturer's protocol

Eukaryotic cell lines

Policy information about cell lines and Sex and Gender in Research

Cell line source(s)

State the source of each cell line used and the sex of all primary cell lines and cells derived from human participants or vertebrate models.

Authentication

Describe the authentication procedures for each cell line used OR declare that none of the cell lines used were authenticated.

Mycoplasma contamination

Confirm that all cell lines tested negative for mycoplasma contamination OR describe the results of the testing for mycoplasma contamination OR declare that the cell lines were not tested for mycoplasma contamination.

Commonly misidentified lines
(See ICLAC register)

Name any commonly misidentified cell lines used in the study and provide a rationale for their use.

Palaeontology and Archaeology

Specimen provenance

Provide provenance information for specimens and describe permits that were obtained for the work (including the name of the issuing authority, the date of issue, and any identifying information). Permits should encompass collection and, where applicable, export.

Specimen deposition

Indicate where the specimens have been deposited to permit free access by other researchers.

Dating methods	*If new dates are provided, describe how they were obtained (e.g. collection, storage, sample pretreatment and measurement), where they were obtained (i.e. lab name), the calibration program and the protocol for quality assurance OR state that no new dates are provided.*

☐ Tick this box to confirm that the raw and calibrated dates are available in the paper or in Supplementary Information.

Ethics oversight	*Identify the organization(s) that approved or provided guidance on the study protocol, OR state that no ethical approval or guidance was required and explain why not.*

Note that full information on the approval of the study protocol must also be provided in the manuscript.

Animals and other research organisms

Policy information about studies involving animals; ARRIVE guidelines recommended for reporting animal research, and Sex and Gender in Research

Laboratory animals	In this study, rattus norvegicus Fischer 344 adult rodents with a weight range of 150-191 grams were used for in vivo studies. Our severe C5 contusion animal model makes animals more prone for urinary tract infections. This is specifically augmented in male rats compared to female rats, due to females having a shorter urethra, making them less prone to bladder infections. Because of this, we find it unethical to subject animal subjects to additional pain and infection and only conducted our in vivo animal studies in female rats.<br>Laboratory mice C57bl/6 adult rodents with a weight range of 17-24 grams were used for in vitro studies, both biological male and female mice were used. A single adult male cynomolgus macaque age 5.5 years was used for in vitro studies.
Wild animals	*Provide details on animals observed in or captured in the field; report species and age where possible. Describe how animals were caught and transported and what happened to captive animals after the study (if killed, explain why and describe method; if released, say where and when) OR state that the study did not involve wild animals.*
Reporting on sex	*Indicate if findings apply to only one sex; describe whether sex was considered in study design, methods used for assigning sex. Provide data disaggregated for sex where this information has been collected in the source data as appropriate; provide overall numbers in this Reporting Summary. Please state if this information has not been collected. Report sex-based analyses where performed, justify reasons for lack of sex-based analysis.*
Field-collected samples	*For laboratory work with field-collected samples, describe all relevant parameters such as housing, maintenance, temperature, photoperiod and end-of-experiment protocol OR state that the study did not involve samples collected from the field.*
Ethics oversight	All animal protocols were approved by the VASDHS Institutional Animal Cara and Use Committee.

Note that full information on the approval of the study protocol must also be provided in the manuscript.

Clinical data

Policy information about clinical studies
All manuscripts should comply with the ICMJE guidelines for publication of clinical research and a completed CONSORT checklist must be included with all submissions.

Clinical trial registration	*Provide the trial registration number from ClinicalTrials.gov or an equivalent agency.*
Study protocol	*Note where the full trial protocol can be accessed OR if not available, explain why.*
Data collection	*Describe the settings and locales of data collection, noting the time periods of recruitment and data collection.*
Outcomes	*Describe how you pre-defined primary and secondary outcome measures and how you assessed these measures.*

Dual use research of concern

Policy information about dual use research of concern

Hazards

Could the accidental, deliberate or reckless misuse of agents or technologies generated in the work, or the application of information presented in the manuscript, pose a threat to:

No	Yes	
☐	☐	Public health
☐	☐	National security
☐	☐	Crops and/or livestock
☐	☐	Ecosystems
☐	☐	Any other significant area

Experiments of concern

Does the work involve any of these experiments of concern:

No	Yes	
☐	☐	Demonstrate how to render a vaccine ineffective
☐	☐	Confer resistance to therapeutically useful antibiotics or antiviral agents
☐	☐	Enhance the virulence of a pathogen or render a nonpathogen virulent
☐	☐	Increase transmissibility of a pathogen
☐	☐	Alter the host range of a pathogen
☐	☐	Enable evasion of diagnostic/detection modalities
☐	☐	Enable the weaponization of a biological agent or toxin
☐	☐	Any other potentially harmful combination of experiments and agents

Plants

Seed stocks	*Report on the source of all seed stocks or other plant material used. If applicable, state the seed stock centre and catalogue number. If plant specimens were collected from the field, describe the collection location, date and sampling procedures.*
Novel plant genotypes	*Describe the methods by which all novel plant genotypes were produced. This includes those generated by transgenic approaches, gene editing, chemical/radiation-based mutagenesis and hybridization. For transgenic lines, describe the transformation method, the number of independent lines analyzed and the generation upon which experiments were performed. For gene-edited lines, describe the editor used, the endogenous sequence targeted for editing, the targeting guide RNA sequence (if applicable) and how the editor was applied.*
Authentication	*Describe any authentication procedures for each seed stock used or novel genotype generated. Describe any experiments used to assess the effect of a mutation and, where applicable, how potential secondary effects (e.g. second site T-DNA insertions, mosiacism, off-target gene editing) were examined.*

ChIP-seq

Data deposition

☐ Confirm that both raw and final processed data have been deposited in a public database such as GEO.

☐ Confirm that you have deposited or provided access to graph files (e.g. BED files) for the called peaks.

Data access links<br>*May remain private before publication.*	*For "Initial submission" or "Revised version" documents, provide reviewer access links. For your "Final submission" document, provide a link to the deposited data.*
Files in database submission	*Provide a list of all files available in the database submission.*
Genome browser session<br>(e.g. UCSC)	*Provide a link to an anonymized genome browser session for "Initial submission" and "Revised version" documents only, to enable peer review. Write "no longer applicable" for "Final submission" documents.*

Methodology

Replicates	*Describe the experimental replicates, specifying number, type and replicate agreement.*
Sequencing depth	*Describe the sequencing depth for each experiment, providing the total number of reads, uniquely mapped reads, length of reads and whether they were paired- or single-end.*
Antibodies	*Describe the antibodies used for the ChIP-seq experiments; as applicable, provide supplier name, catalog number, clone name, and lot number.*
Peak calling parameters	*Specify the command line program and parameters used for read mapping and peak calling, including the ChIP, control and index files used.*

Data quality	*Describe the methods used to ensure data quality in full detail, including how many peaks are at FDR 5% and above 5-fold enrichment.*
Software	*Describe the software used to collect and analyze the ChIP-seq data. For custom code that has been deposited into a community repository, provide accession details.*

Flow Cytometry

Plots

Confirm that:

☐ The axis labels state the marker and fluorochrome used (e.g. CD4-FITC).

☐ The axis scales are clearly visible. Include numbers along axes only for bottom left plot of group (a 'group' is an analysis of identical markers).

☐ All plots are contour plots with outliers or pseudocolor plots.

☐ A numerical value for number of cells or percentage (with statistics) is provided.

Methodology

Sample preparation	*Describe the sample preparation, detailing the biological source of the cells and any tissue processing steps used.*
Instrument	*Identify the instrument used for data collection, specifying make and model number.*
Software	*Describe the software used to collect and analyze the flow cytometry data. For custom code that has been deposited into a community repository, provide accession details.*
Cell population abundance	*Describe the abundance of the relevant cell populations within post-sort fractions, providing details on the purity of the samples and how it was determined.*
Gating strategy	*Describe the gating strategy used for all relevant experiments, specifying the preliminary FSC/SSC gates of the starting cell population, indicating where boundaries between "positive" and "negative" staining cell populations are defined.*

☐ Tick this box to confirm that a figure exemplifying the gating strategy is provided in the Supplementary Information.

Magnetic resonance imaging

Experimental design

Design type	*Indicate task or resting state; event-related or block design.*
Design specifications	*Specify the number of blocks, trials or experimental units per session and/or subject, and specify the length of each trial or block (if trials are blocked) and interval between trials.*
Behavioral performance measures	*State number and/or type of variables recorded (e.g. correct button press, response time) and what statistics were used to establish that the subjects were performing the task as expected (e.g. mean, range, and/or standard deviation across subjects).*

Acquisition

Imaging type(s)	*Specify: functional, structural, diffusion, perfusion.*
Field strength	*Specify in Tesla*
Sequence & imaging parameters	*Specify the pulse sequence type (gradient echo, spin echo, etc.), imaging type (EPI, spiral, etc.), field of view, matrix size, slice thickness, orientation and TE/TR/flip angle.*
Area of acquisition	*State whether a whole brain scan was used OR define the area of acquisition, describing how the region was determined.*

Diffusion MRI ☐ Used ☐ Not used

Preprocessing

Preprocessing software	*Provide detail on software version and revision number and on specific parameters (model/functions, brain extraction, segmentation, smoothing kernel size, etc.).*
Normalization	*If data were normalized/standardized, describe the approach(es): specify linear or non-linear and define image types used for transformation OR indicate that data were not normalized and explain rationale for lack of normalization.*

Normalization template	*Describe the template used for normalization/transformation, specifying subject space or group standardized space (e.g. original Talairach, MNI305, ICBM152) OR indicate that the data were not normalized.*
Noise and artifact removal	*Describe your procedure(s) for artifact and structured noise removal, specifying motion parameters, tissue signals and physiological signals (heart rate, respiration).*
Volume censoring	*Define your software and/or method and criteria for volume censoring, and state the extent of such censoring.*

Statistical modeling & inference

Model type and settings	*Specify type (mass univariate, multivariate, RSA, predictive, etc.) and describe essential details of the model at the first and second levels (e.g. fixed, random or mixed effects; drift or auto-correlation).*
Effect(s) tested	*Define precise effect in terms of the task or stimulus conditions instead of psychological concepts and indicate whether ANOVA or factorial designs were used.*

Specify type of analysis: ☐ Whole brain ☐ ROI-based ☐ Both

Statistic type for inference (See Eklund et al. 2016)	*Specify voxel-wise or cluster-wise and report all relevant parameters for cluster-wise methods.*
Correction	*Describe the type of correction and how it is obtained for multiple comparisons (e.g. FWE, FDR, permutation or Monte Carlo).*

Models & analysis

n/a	Involved in the study
☐	☐ Functional and/or effective connectivity
☐	☐ Graph analysis
☐	☐ Multivariate modeling or predictive analysis

Functional and/or effective connectivity	*Report the measures of dependence used and the model details (e.g. Pearson correlation, partial correlation, mutual information).*
Graph analysis	*Report the dependent variable and connectivity measure, specifying weighted graph or binarized graph, subject- or group-level, and the global and/or node summaries used (e.g. clustering coefficient, efficiency, etc.).*
Multivariate modeling and predictive analysis	*Specify independent variables, features extraction and dimension reduction, model, training and evaluation metrics.*
